# Supplementary material for: Protocol for a scoping review to identify research reporting on eating disorders in minority ethnic populations in the UK, Canada, Australia and New Zealand
Source: BMJ Open. 2024 Feb 13;14(2):e075034. doi: 10.1136/bmjopen-2023-075034 (PMC10868291; doi:10.1136/bmjopen-2023-075034)
Supplement: Author's manuscript [file bmjopen-2023-075034.draft_revisions.pdf]

BMJ Open is committed to open peer review. As part of this commitment we make the peer review history of every article we publish publicly available.

When an article is published we post the peer reviewers' comments and the authors' responses online. We also post the versions of the paper that were used during peer review. These are the versions that the peer review comments apply to.

The versions of the paper that follow are the versions that were submitted during the peer review process. They are not the versions of record or the final published versions. They should not be cited or distributed as the published version of this manuscript.

BMJ Open is an open access journal and the full, final, typeset and author-corrected version of record of the manuscript is available on our site with no access controls, subscription charges or pay-per-view fees (http://bmjopen.bmj.com).

If you have any questions on BMJ Open's open peer review process please email info.bmjopen@bmj.com

BMJ Open

# A protocol for a scoping review to identify research reporting ethnic variation in eating disorders and access to treatment

| | |
|---|---|
| Journal: | *BMJ Open* |
| Manuscript ID | bmjopen-2023-075034 |
| Article Type: | Protocol |
| Date Submitted by the Author: | 25-Apr-2023 |
| Complete List of Authors: | Tuomainen, Helena; University of Warwick<br>McGowan, Rose; University of Warwick<br>Guy, Katie; Birmingham Women's and Children's Hospitals NHS Foundation Trust<br>McNeil, Sheryllin; Birmingham Women's and Children's Hospitals NHS Foundation Trust |
| Keywords: | Eating disorders < PSYCHIATRY, Health Services Accessibility, Systematic Review |
| | |

SCHOLARONE™
Manuscripts

# A protocol for a scoping review to identify research reporting ethnic variation in eating disorders and access to treatment

Tuomainen H[1], McGowan R[1], Guy K[2], McNeil S[2]

[1]Warwick Medical School, University of Warwick, Coventry, UK

[2]Birmingham Women's and Children's NHS Foundation Trust, Birmingham, UK

helena.tuomainen@warwick.ac.uk

Rose.McGowan@warwick.ac.uk

katie.guy@nhs.net

sheryllin.mcneil1@nhs.net

Corresponding author:

Helena Tuomainen
Warwick Medical School
University of Warwick
CV47AL
helena.tuomainen@warwick.ac.uk
+44-07722-330979

Keywords: Eating disorders, Population groups, Ethnicity, treatment, healthcare disparities

For peer review only

# Abstract

### Introduction

Eating disorders are common, remain life threatening and are becoming increasingly reported and diagnosed. However, there is a disparity in the reporting of the relationship between eating disorders and ethnicity, with some research finding lower prevalence and treatment of eating disorders among ethnic minorities; other research reports higher prevalence of some eating disorders among ethnic minorities in comparison to white counterparts. Further still there is discussion and debate about the reason for the disparities found; some researchers propose that ethnic minorities are less likely to experience eating disorders, while others argue that these populations are not adequately identified or treated within eating disorder services. Our scoping review aims to examine the extent, range and nature of research activity into eating disorders and ethnicity with a special focus on ethnic variations in the prevalence, access to and experience of treatment in eating disorders.

### Methods and Analysis

Arksey and O'Malley's scoping review methodology framework will guide the conduct of this scoping review. A detailed search strategy will be employed to search Medline, EMBASE, PsycInfo, CINAHL, and Web of Science databases. Specific inclusion and exclusion criteria will be applied and two reviewers will independently screen all abstracts and full-text studies for inclusion in order to capture the most relevant literature. The data will then be extracted and analysed, to ultimately be summarised in a way that maps the breadth of the literature surrounding ethnic variation in eating disorders and identifies gaps or limitations.

### Ethics and Dissemination

No ethical approval is required for this study. Findings will be disseminated via conference presentations and the submission of the final review for peer-reviewed publication.


# Strengths and limitations of this study

- This will be the first scoping review to examine eating disorders within ethnic minority populations.
- This protocol will ensure transparency in methodology and reduce the likelihood of reviewing bias.
- There will be no quality assessment of the included studies.



For peer review only - http://bmjopen.bmj.com/site/about/guidelines.xhtml

For peer review only

- Only studies published in English will be included.

# Introduction

## Background

Eating disorders (EDs) are complex psychiatric conditions characterised by disordered eating and/or weight-control behaviours[1] which typically have their onset during adolescence[2]. A recent systematic review reported pooled lifetime and 12-month prevalence of eating disorders of 0.91% and 0.43%, respectively[3]. EDs can cause devastating effects on the health and lives of those affected; they have high co-morbidity with other psychiatric disorders, including suicidal thoughts and attempts[4 5]. The medical complications that often accompany EDs involve almost all organ systems and can be life-threatening[6]. Anorexia nervosa and bulimia nervosa are associated with significantly increased mortality rates[7]. Given the negative consequences of EDs, it is imperative that we understand the prevalence and treatment within and across ethnic groups.

EDs are recognised as affecting all ethnic and racial groups[8], despite a prevailing historic stereotype of EDs only affecting "skinny, white, affluent girls" (the SWAG stereotype [9]). There is a large disparity in research about ethnicity and EDs, with some studies reporting no differences in prevalence of symptoms across different ethnic groups[10], some finding that white students were more likely to have an ED diagnosis than non-white students[9], and others finding a higher prevalence of disordered eating amongst ethnic minority men and women in comparison to White peers[11]. Further, Waller et al[12] reported that referrals to an eating disorder service within a multi-ethnic urban area did not reflect the ethnic variation of the local population, with ethnic minority patients less likely to be referred to the services than white patients. Evidently, our understanding of the relationship between EDs and ethnicity is weak. Similarly, some researchers have found that White adults have a higher lifetime prevalence of EDs when compared to other ethnic groups[13 14], whereas in contrast others have reported no significant differences in prevalence of EDs[15 16].

Despite similar, and in some cases increased rates of EDs amongst ethnic minority groups, in their systematic review Sinha and Warfa[17] reported ethnic minority individuals in both the UK and US were less likely to seek and receive treatment, as well as less frequently referred to specialist ED services by their GP. Since publication of this review further studies have replicated these findings. Coffino, Udo[18] found ethnic differences in help-seeking behaviours. Men and non-Hispanic blacks and Hispanics were less likely to seek for help for binge eating disorder than women or non-Hispanic whites and Hispanics less likely to seek help for anorexia nervosa relative to non-Hispanic whites. Solmi et al



For peer review only - http://bmjopen.bmj.com/site/about/guidelines.xhtml

[19], who identified similar findings of lower rates of help-seeking/treatment despite higher prevalence rates among ethnic minority populations, speculated that this could reflect these being less severe cases, or that there is a failure to diagnose in these populations. Sonneville and Lipson[9] suggested three causes of this failure to receive treatment for an ED for those that do not fit the SWAG stereotype: lack of perceived need for treatment, failure by clinicians to make appropriate diagnosis and/or referral, and lack of treatment resources.

There have been a number of qualitative interview and focus group studies exploring experiences of seeking help or treatment for an ED or disordered eating amongst ethnic minority groups from the perspective of service users, caregivers and clinicians[20 21, 22 23 24 25]. Collectively these studies suggest there a number of barriers common across all ethnicities to help-seeking for EDs/disordered eating including stigma and shame, failure to recognise illness severity, practical barriers, negative treatment attitudes, lack of support and encouragement and ambivalence to change.

However, there were also some barriers which appeared unique to ethnic minorities including racial stereotypes held by clinicians[22], family living arrangements[25], confidentiality concerns[22 25] and communication/language difficulties[24]. Further, it seemed the experience of shame and stigma for those from Black and Asian backgrounds appeared qualitatively different from non-minority peers and was rooted in and perpetuated by cultural and religious beliefs and denial of mental health problems in older generations [21 22 23 24 25]. These barriers in part may explain the reduced presentation of ethnic minorities to ED services. It is of note however, that most of these qualitative studies focused on Asian minority groups specifically and did not look into whether there may be disorder-specific barriers.

## Rationale

The negative consequences of EDs combined with the disparity in current literature surrounding EDs and ethnic groups necessitate a review of the existing research to establish what is known about EDs and ethnic variation. The impact of ethnic minority status needs to be understood to appropriately identify and understand barriers that may affect access to services and treatment as a whole, not only those that fit the stereotype, and for providing the most suitable treatment options, including the development and delivery of culturally sensitive treatment. The present scoping review will map the research foci in ethnic variation in eating disorders, with a special focus on ethnic variations in the prevalence, timely access to services and treatment, and proceed to identify current gaps in literature. This information will provide a basis for future research investigating ethnic variation in eating disorders.



For peer review only - http://bmjopen.bmj.com/site/about/guidelines.xhtml

For peer review only

A preliminary search of MEDLINE, PROSPERO, the Cochrane Database of Systematic Reviews and *JBI Evidence Synthesis* was conducted and no current or underway systematic reviews or scoping reviews on the topic were identified. To our knowledge, there is no published synthesis of eating disorder and ethnicity literature using scoping review methodology.

## Objectives

In this scoping literature review we aim to examine the extent, range and nature of research activity into ethnic variation in eating disorders. The specific objectives are to:

- Conduct a systematic search of literature concerning eating disorders and ethnicity
- Map the literature according to study themes, target populations, methodologies, and findings regarding prevalence, access to and experience of treatment
- Identify gaps or limitations in current existing literature

# Methods and Analysis

A scoping review was selected as the methodology because it is a useful way to systematically map the existing literature on a topic and give clear indication of volume and an overview of their focus. In line with Munn et al[26], a scoping review is the methodology of choice for identifying the types of available evidence in the field, to examine how research is conducted in the field, and to identify knowledge gaps. This scoping review will utilise the five stages set out by Arksey and O'Malley[20]: (1) Identifying the research questions; (2) identifying the relevant literature; (3) selecting studies; (4) charting the data; and (5) collating, summarising and reporting the results.

## Stage 1: Identifying the Research Questions

The main, overarching research question for this scoping review has been identified: 'What does current research report on the ethnic variations in the prevalence, access to services and experience of treatment in Eating Disorders'. This broad question will allow us to examine the full breadth of existing literature and to guide or modify our research questions as part of the iterative process as we become more familiar with the existing literature. To complete and complement the research question, we have identified several secondary questions to guide the reviewing process. These will include, but are not limited to: (1) What topics are being addressed? (2) What types of research have been conducted? (3) In which settings have studies been conducted? (4) Which populations have been targeted for this research? (5) What eating disorders have been researched? (6) Is intervention effectiveness by ethnic group evident? (7) What are the gaps?



For peer review only - http://bmjopen.bmj.com/site/about/guidelines.xhtml

For peer review only

## Stage 2: Identifying Relevant Studies/Literature

The second stage of the review will include identifying relevant studies for selection and data extraction. This will involve a systematic search strategy of the following electronic databases: (1) Medline (2) PsycINFO; (3) EMBASE; (4) CINAHL and (5) Web of Science. Reference lists of included studies will be hand-searched to identify additional studies of relevance.

No date limits will be applied to the search results, but the database search query will be set to produce articles in English only. With input from the research team and in consultation with a specialty academic support librarian, we developed a Medline search strategy which generated 1312 results. The proposed search strategy is shown in the online supplementary appendix 1. This search will be translated and tailored for use in the PsycINFO, EMBASE, CINAHL and Web of Science databases. In order to develop a comprehensive review of existing research and clinical guidelines related to ethnic variation in eating disorders, both empirical (primary studies) and grey literature (conference abstracts and PhD theses) will be included in our search. Upon completion, the searches from each database will be documented and references will be imported into the citation management system Rayyan, where duplicates will automatically be eliminated before studies are screened for inclusion.

This scoping review will consider for inclusion primary research and secondary research with any study design,  including experimental and quasi-experimental study designs and descriptive and analytical observational studies, and qualitative study designs.

## Stage 3: Selecting Studies

The process of selecting studies will consist of two screenings according to the inclusion and exclusion criteria. Each screening will involve two data reviewers (RM, HT) independently screening all relevant studies identified. The first screening process will consist of only title and abstract, and the number of included and excluded will be recorded and decisions will be made as follows: (1) if both reviewers agree to include, the study will move on to the second screening; (2) if both reviewers agree to exclude, the study will not be read in full and will be excluded from the review; (3) if the reviewers are not unanimous in their agreement, the study will also move onto the second screening to be read in full before a final decision is made.

The second screening will involve the two data reviewers independently reading the remaining articles in full to determine whether they meet the inclusion and exclusion criteria. If the decision to include or exclude if not unanimous, both reviewers will assess the study together and discuss disagreements. A third review may be consulted for arbitration of any conflicting decisions at this point.



For peer review only - http://bmjopen.bmj.com/site/about/guidelines.xhtml
For peer review only

| Inclusion Criteria | Exclusion Criteria |
|---|---|
| - Reports on EDs and ethnicity<br>- Presents findings according to ethnic groups<br>- Ethnic minority of that country<br>- Primary/secondary research<br>- Any study design<br>- Any country<br>- Published in English | - No ethnicity-based findings reported<br>- Solely ethnic majorities<br>- Does not include/focused on EDs<br>- Does not include findings for ethnic minorities<br>- Systematic reviews<br>- Commentaries<br>- Editorials |

## Stage 4: Charting the Data/Data Extraction

Once studies have been identified for final inclusion, data from these studies will be collected and charted. Data to be extracted will include some standard information (such as author, year of publication, country of publication, sample size, study objectives) and additional information specific to ethnic variation within eating disorders according to our primary and secondary research questions. This will include population characteristics and demographics (such as ED diagnosis, ethnicity), study design, setting in which the study was conducted, summary of findings, and recommendations of the author(s) if applicable. Additional categories of interest may emerge during this process. The research team will meet regularly to discuss progress and at this point it can be discussed if additional data extraction categories are needed, or if missing data emerge. Any changes will be reported with the findings.

## Stage 5: Collating, Summarising and Reporting the Results

As the purpose of a scoping review is to aggregate the findings and present an overview, rather than a meta-synthesis, all data collected will be summarised in a way that maps the breadth of the literature surrounding ethnic variation in eating disorders with a special focus on ethnic variations in prevalence, timely access to services and treatment in eating disorders. Studies will be classified and grouped according to their characteristics as noted during data extraction and charting. The data will be presented in tables, charts and visual maps to provide an overview of the current literature. If data and resources permit, qualitative studies will be analysed using thematic analysis. We will then be able to identify gaps in the research and determine where more in-depth analysis is required.



For peer review only - http://bmjopen.bmj.com/site/about/guidelines.xhtml

# Ethics and Dissemination

Scoping reviews involve methodological examination and integration of existing literature and resources therefore this study does not require ethical approval.

Once all stages of the scoping review are complete, findings will be disseminated via conference presentations and the submission of the final review for peer-reviewed publication.

This study will provide a high-quality summary of available literature on the topic of EDs and ethnicity. The scoping review produced will provide identification of gaps in knowledge and research in relation to EDs and ethnicity, and may make suggestions for future direction(s) of research in the area. The highlighted gaps may assist researchers and policy makers to design new research questions and interventions to improve ED treatment for all.

## Patient and Public Involvement

Patients or the public have not been involved in the design, or conduct, or reporting, or dissemination plans of our research.

# Acknowledgements

The authors thank Samantha Johnson, Academic Support Librarian, for assisting in the search strategy.

# Author Contributions

HT and SM conceived the idea, developed the research question, and contributed meaningfully to the drafting and editing; HT was responsible for the design of the work and methods; RM aided developing the research question and study methods and drafted the first manuscript; KG contributed meaningfully to the drafting and editing. All authors approved the final manuscript.

# Funding Statement

This research is funded by the National Institute for Health and Care Research (NIHR) Applied Research Collaboration (ARC) West Midlands. The views expressed are those of the authors and not necessarily those of the NIHR or the Department of Health and Social Care.

For peer review only

## Competing Interests Statement

None declared.

## Word Count

2066

For peer review only



For peer review only - http://bmjopen.bmj.com/site/about/guidelines.xhtml

# References

1. American Psychiatric Association D, Association AP. Diagnostic and statistical manual of mental disorders: DSM-5: American psychiatric association Washington, DC; 2013.

2. Volpe U, Tortorella A, Manchia M, Monteleone AM, Albert U, Monteleone P. Eating disorders: What age at onset? Psychiatry Research. 2016;238:225-7.

3. Qian J, Wu Y, Liu F, Zhu Y, Jin H, Zhang H, et al. An update on the prevalence of eating disorders in the general population: a systematic review and meta-analysis. Eating and Weight Disorders-Studies on Anorexia, Bulimia and Obesity. 2021:1-14.

4. Chamay-Weber C, Narring F, Michaud P-A. Partial eating disorders among adolescents: A review. Journal of adolescent health. 2005;37(5):416-26.

5. Lewinsohn PM, Striegel-Moore RH, Seeley JR. Epidemiology and natural course of eating disorders in young women from adolescence to young adulthood. Journal of the American Academy of Child & Adolescent Psychiatry. 2000;39(10):1284-92.

6. Voderholzer U, Haas V, Correll CU, Körner T. Medical management of eating disorders: an update. Current opinion in psychiatry. 2020;33(6):542-53.

7. van Hoeken D, Hoek HW. Review of the burden of eating disorders: mortality, disability, costs, quality of life, and family burden. Current Opinion in Psychiatry. 2020;33(6):521.

8. Rodgers RF, Berry R, Franko DL. Eating disorders in ethnic minorities: An update. Current psychiatry reports. 2018;20:1-11.

9. Sonneville K, Lipson S. Disparities in eating disorder diagnosis and treatment according to weight status, race/ethnicity, socioeconomic background, and sex among college students. International Journal of Eating Disorders. 2018;51(6):518-26.

10. Cheng ZH, Perko VL, Fuller-Marashi L, Gau JM, Stice E. Ethnic differences in eating disorder prevalence, risk factors, and predictive effects of risk factors among young women. Eating behaviors. 2019;32:23-30.



For peer review only - http://bmjopen.bmj.com/site/about/guidelines.xhtml

11. Simone M, Telke S, Anderson LM, Eisenberg M, Neumark-Sztainer D. Ethnic/racial and gender differences in disordered eating behavior prevalence trajectories among women and men from adolescence into adulthood. Social Science & Medicine. 2022;294:114720.

12. Waller G, Schmidt U, Treasure J, Emanuelli F, Alenya J, Crockett J, et al. Ethnic origins of patients attending specialist eating disorders services in a multiethnic urban catchment area in the United Kingdom. International Journal of Eating Disorders. 2009;42(5):459-63.

13. Udo T, Grilo CM. Prevalence and correlates of DSM-5–defined eating disorders in a nationally representative sample of US adults. Biological psychiatry. 2018;84(5):345-54.

14. Striegel-Moore RH, Dohm FA, Kraemer HC, Taylor CB, Daniels S, Crawford PB, et al. Eating disorders in white and black women. American Journal of Psychiatry. 2003;160(7):1326-31.

15. Solmi F, Hotopf M, Hatch S, Treasure J, Micali N. Eating disorders in a multi-ethnic inner-city UK sample: prevalence, comorbidity and service use. Social Psychiatry and Psychiatric Epidemiology. 2016;51:369-81.

16. Marques L, Alegria M, Becker AE, Chen Cn, Fang A, Chosak A, et al. Comparative prevalence, correlates of impairment, and service utilization for eating disorders across US ethnic groups: Implications for reducing ethnic disparities in health care access for eating disorders. International Journal of Eating Disorders. 2011;44(5):412-20.

17. Sinha S, Warfa N. Treatment of eating disorders among ethnic minorities in western settings: a systematic review. Psychiatria Danubina. 2013;25(suppl 2):295-9.

18. Coffino JA, Udo T, Grilo CM, editors. Rates of help-seeking in US adults with lifetime DSM-5 eating disorders: prevalence across diagnoses and differences by sex and ethnicity/race. Mayo Clinic Proceedings; 2019: Elsevier.

19. Solmi F, Hatch SL, Hotopf M, Treasure J, Micali N. Prevalence and correlates of disordered eating in a general population sample: the South East London Community Health (SELCoH) study. Social psychiatry and psychiatric epidemiology. 2014;49:1335-46.



For peer review only - http://bmjopen.bmj.com/site/about/guidelines.xhtml

20. Arksey H, O'Malley L. Scoping studies: towards a methodological framework. International journal of social research methodology. 2005;8(1):19-32.

21. Channa S, Lavis A, Connor C, Palmer C, Leung N, Birchwood M. Overlaps and disjunctures: a cultural case study of a british indian young woman's experiences of bulimia nervosa. Culture, Medicine, and Psychiatry. 2019;43:361-86.

22. Chowbey P, Salway S, Ismail M. Influences on diagnosis and treatment of eating disorders among minority ethnic people in the UK. Journal of Public Mental Health. 2012.

23. Javier SJ, Belgrave FZ. "I'm not White, I have to be pretty and skinny": A qualitative exploration of body image and eating disorders among Asian American women. Asian American Journal of Psychology. 2019;10(2):141.

24. Kanakam N. Therapists' Experiences of Working with Ethnic Minority Females with Eating Disorders: A Qualitative Study. Culture, Medicine, and Psychiatry. 2022;46(2):414-34.

25. Wales J, Brewin N, Raghavan R, Arcelus J. Exploring barriers to South Asian help-seeking for eating disorders. Mental Health Review Journal. 2017;22(1):40-50.

26. Munn Z, Peters MD, Stern C, Tufanaru C, McArthur A, Aromataris E. Systematic review or scoping review? Guidance for authors when choosing between a systematic or scoping review approach. BMC medical research methodology. 2018;18:1-7.

For peer review only



For peer review only - http://bmjopen.bmj.com/site/about/guidelines.xhtml

## Search strategy

Example search strategy for Medline.

1 "feeding and eating disorders"/ or anorexia nervosa/ or avoidant restrictive food intake disorder/ or binge-eating disorder/ or bulimia nervosa/ or diabulimia/ or "feeding and eating disorders of childhood"/ or food addiction/ or night eating syndrome/ or orthorexia nervosa/ or pica/ or relative energy deficiency in sport/ or female athlete triad syndrome/ or rumination syndrome/ 35804

2 eating disorder*.mp. 33463

3 anorexi*.mp. 42181

4 bulimi*.mp. 12324

5 ARFID.mp. 268

6 avoidant restrictive food intake disorder.mp. 432

7 binge eating.mp. 7407

8 pica.mp. 3495

9 rumination disorder.mp. 41

10 rumination syndrome.mp. 162

11 1 or 2 or 3 or 4 or 5 or 6 or 7 or 8 or 9 or 10 73164

12 population groups/ or african people/ or north african people/ or sub-saharan african people/ or central african people/ or east african people/ or southern african people/ or west african people/ or asian people/ or asian/ or central asian people/ or east asian people/ or north asian people/ or southeast asian people/ or west asian people/ or middle eastern people/ or south asian people/ or black people/ or "black or african american"/ or caribbean people/ or central american people/ or indians, central american/ or european people/ or eastern european people/ or "scandinavians and nordic people"/ or "middle eastern and north africans"/ or north american people/ or "american indian or alaska native"/ or indians, north american/ or alaskan natives/ or indigenous canadians/ or inuit/ or navajo people/ or pima people/ or population groups, us/ or ethnicity/ or "hispanic or latino"/ or mexican americans/ or racial groups/ or "native hawaiian or other pacific islander"/ or white/ or oceanians/ or australasian people/ or "australian aboriginal and torres strait islander peoples"/ or pacific island people/ or maori people/ or south american people/ or indians, south american/ or white people/326840

For peer review only

13 "emigrants and immigrants"/ or undocumented immigrants/ or refugees/ or "transients and migrants"/ 39912

14 "black or african american"/ or amish/ or arabs/ or "asian american native hawaiian and pacific islander"/ or asian/ or "native hawaiian or other pacific islander"/ or "hispanic or latino"/ or mexican americans/ or indigenous peoples/ or "american indian or alaska native"/ or indians, central american/ or indians, north american/ or alaskan natives/ or indigenous canadians/ or inuit/ or navajo people/ or pima people/ or indians, south american/ or "australian aboriginal and torres strait islander peoples"/ or maori people/ or roma/ 133629

15 asia, southern/ or afghanistan/ or bangladesh/ or bhutan/ or india/ or sikkim/ or maldives/ or nepal/ or pakistan/ or sri lanka/ 172233

16 ethnic minorit*.mp. 15411

17 racial minorit*.mp. 2337

18 multi-ethnic*.mp. 5370

19 "ethnic and racial minorities"/ or minority groups/ 17800

20 BAME.mp. 358

21 BIPOC.mp. 264

22 people of colo*r.mp. 1373

23 12 or 13 or 14 or 15 or 16 or 17 or 18 or 19 or 20 or 21 or 22 549059

24 11 and 23 1312

For peer review only

## Preferred Reporting Items for Systematic reviews and Meta-Analyses extension for Scoping Reviews (PRISMA-ScR) Checklist

| SECTION | ITEM | PRISMA-ScR CHECKLIST ITEM | REPORTED ON PAGE # |
|---|---|---|---|
| **TITLE** | | | |
| Title | 1 | Identify the report as a scoping review. | |
| **ABSTRACT** | | | |
| Structured summary | 2 | Provide a structured summary that includes (as applicable): background, objectives, eligibility criteria, sources of evidence, charting methods, results, and conclusions that relate to the review questions and objectives. | |
| **INTRODUCTION** | | | |
| Rationale | 3 | Describe the rationale for the review in the context of what is already known. Explain why the review questions/objectives lend themselves to a scoping review approach. | |
| Objectives | 4 | Provide an explicit statement of the questions and objectives being addressed with reference to their key elements (e.g., population or participants, concepts, and context) or other relevant key elements used to conceptualize the review questions and/or objectives. | |
| **METHODS** | | | |
| Protocol and registration | 5 | Indicate whether a review protocol exists; state if and where it can be accessed (e.g., a Web address); and if available, provide registration information, including the registration number. | |
| Eligibility criteria | 6 | Specify characteristics of the sources of evidence used as eligibility criteria (e.g., years considered, language, and publication status), and provide a rationale. | |
| Information sources* | 7 | Describe all information sources in the search (e.g., databases with dates of coverage and contact with authors to identify additional sources), as well as the date the most recent search was executed. | |
| Search | 8 | Present the full electronic search strategy for at least 1 database, including any limits used, such that it could be repeated. | |
| Selection of sources of evidence† | 9 | State the process for selecting sources of evidence (i.e., screening and eligibility) included in the scoping review. | |
| Data charting process‡ | 10 | Describe the methods of charting data from the included sources of evidence (e.g., calibrated forms or forms that have been tested by the team before their use, and whether data charting was done independently or in duplicate) and any processes for obtaining and confirming data from investigators. | |
| Data items | 11 | List and define all variables for which data were sought and any assumptions and simplifications made. | |
| Critical appraisal of individual sources of evidence§ | 12 | If done, provide a rationale for conducting a critical appraisal of included sources of evidence; describe the methods used and how this information was used in any data synthesis (if appropriate). | |
| Synthesis of results | 13 | Describe the methods of handling and summarizing the data that were charted. | |

For peer review only

| SECTION | ITEM | PRISMA-ScR CHECKLIST ITEM | REPORTED ON PAGE # |
|---|---|---|---|
| **RESULTS** | | | |
| Selection of sources of evidence | 14 | Give numbers of sources of evidence screened, assessed for eligibility, and included in the review, with reasons for exclusions at each stage, ideally using a flow diagram. | |
| Characteristics of sources of evidence | 15 | For each source of evidence, present characteristics for which data were charted and provide the citations. | |
| Critical appraisal within sources of evidence | 16 | If done, present data on critical appraisal of included sources of evidence (see item 12). | |
| Results of individual sources of evidence | 17 | For each included source of evidence, present the relevant data that were charted that relate to the review questions and objectives. | |
| Synthesis of results | 18 | Summarize and/or present the charting results as they relate to the review questions and objectives. | |
| **DISCUSSION** | | | |
| Summary of evidence | 19 | Summarize the main results (including an overview of concepts, themes, and types of evidence available), link to the review questions and objectives, and consider the relevance to key groups. | |
| Limitations | 20 | Discuss the limitations of the scoping review process. | |
| Conclusions | 21 | Provide a general interpretation of the results with respect to the review questions and objectives, as well as potential implications and/or next steps. | |
| **FUNDING** | | | |
| Funding | 22 | Describe sources of funding for the included sources of evidence, as well as sources of funding for the scoping review. Describe the role of the funders of the scoping review. | |

JBI = Joanna Briggs Institute; PRISMA-ScR = Preferred Reporting Items for Systematic reviews and Meta-Analyses extension for Scoping Reviews.

* Where *sources of evidence* (see second footnote) are compiled from, such as bibliographic databases, social media platforms, and Web sites.

† A more inclusive/heterogeneous term used to account for the different types of evidence or data sources (e.g., quantitative and/or qualitative research, expert opinion, and policy documents) that may be eligible in a scoping review as opposed to only studies. This is not to be confused with *information sources* (see first footnote).

‡ The frameworks by Arksey and O'Malley (6) and Levac and colleagues (7) and the JBI guidance (4, 5) refer to the process of data extraction in a scoping review as data charting.

§ The process of systematically examining research evidence to assess its validity, results, and relevance before using it to inform a decision. This term is used for items 12 and 19 instead of "risk of bias" (which is more applicable to systematic reviews of interventions) to include and acknowledge the various sources of evidence that may be used in a scoping review (e.g., quantitative and/or qualitative research, expert opinion, and policy document).

*From:* Tricco AC, Lillie E, Zarin W, O'Brien KK, Colquhoun H, Levac D, et al. PRISMA Extension for Scoping Reviews (PRISMAScR): Checklist and Explanation. Ann Intern Med. 2018;169:467–473. doi: 10.7326/M18-0850.


For peer review only - http://bmjopen.bmj.com/site/about/guidelines.xhtml

BMJ Open

# A protocol for a scoping review to identify research reporting on eating disorders in minority ethnic populations in the UK, Canada, Australia, and New Zealand

| | |
|---|---|
| Journal: | *BMJ Open* |
| Manuscript ID | bmjopen-2023-075034.R1 |
| Article Type: | Protocol |
| Date Submitted by the Author: | 18-Dec-2023 |
| Complete List of Authors: | Tuomainen, Helena; University of Warwick<br>McGowan, Rose; University of Warwick<br>Williams-Ridgway, Aliyah; University of Warwick<br>Guy, Katie; Birmingham Women's and Children's Hospitals NHS Foundation Trust<br>McNeil, Sheryllin; Birmingham Women's and Children's Hospitals NHS Foundation Trust |
| <b>Primary Subject Heading</b>: | Mental health |
| Secondary Subject Heading: | Health services research |
| Keywords: | Eating disorders < PSYCHIATRY, Health Services Accessibility, Systematic Review |
| | |

SCHOLARONE™
Manuscripts

# A protocol for a scoping review to identify research reporting on eating disorders in minority ethnic populations in the UK, Canada, Australia, and New Zealand

Tuomainen H[1], McGowan R[1], Williams-Ridgway A[1], Guy K[2], McNeil S[2]

[1]Warwick Medical School, University of Warwick, Coventry, UK

[2]Birmingham Women's and Children's NHS Foundation Trust, Birmingham, UK

helena.tuomainen@warwick.ac.uk

Rose.McGowan@warwick.ac.uk

aliyah.williams-ridgway@warwick.ac.uk

katie.guy@nhs.net

sheryllin.mcneil1@nhs.net

Corresponding author:

Helena Tuomainen
Warwick Medical School
University of Warwick
CV47AL
helena.tuomainen@warwick.ac.uk
+44-07722-330979

Keywords: Eating disorders, Population groups, Ethnicity, treatment, healthcare disparities



For peer review only - http://bmjopen.bmj.com/site/about/guidelines.xhtml

# Abstract

### *Introduction*

Eating disorders (EDs) are common, severe and often life-threatening psychiatric conditions. Notwithstanding stereotypes, EDs affect individuals from all racial and ethnic backgrounds. However, despite similar and in some cases increased prevalence of disordered eating and EDs among minority ethnic groups there appear to be disparities when it comes to ED diagnosis and treatment. To date, most of the existing literature exploring disordered eating and EDs among minority ethnic groups has been conducted in the United States (US). The present scoping review aims to examine the extent, range and nature of research activity into disordered eating and EDs in the UK, Canada, Australia and New Zealand providing a comprehensive overview of the existing literature. A special focus will be placed on studies exploring prevalence, access to care and treatment experiences.

### *Methods and Analysis*

The scoping review framework first outlined by Arksey and O'Malley (2005) and improved upon by Levac et al. (2010) was used to guide the development of this scoping review protocol. A detailed systematic search of relevant databases (Medline, EMBASE, PsycInfo, CINAHL, and Web of Science) will be conducted. Relevant literature will also be identified from the reference lists of included studies. Two reviewers will independently screen titles and abstracts and full-text studies for inclusion using specified inclusion and exclusion criteria. A third reviewer will resolve disagreements if necessary. Relevant data will be extracted using a data charting form. Quantitative and qualitative summaries of extracted data will be provided.

### *Ethics and Dissemination*

No ethical approval is required for this study. Findings will be of benefit to researchers, clinicians and policy makers by highlighting areas for future research and identifying ways to making ED treatment more accessible for individuals from all backgrounds. Findings will be disseminated via conferences, presentations and peer-reviewed journals.


# Strengths and limitations of this study

- This will be the first scoping review to examine disordered eating and EDs within minority ethnic populations in the UK, Canada, Australia and New Zealand.
- This protocol will ensure transparency in methodology to reduce the likelihood of reviewing bias.



For peer review only - http://bmjopen.bmj.com/site/about/guidelines.xhtml

- Though EDs are not limited to US and other Western countries, studies conducted in non-Western, low- or middle-income countries are not included in this review.
- There will be no quality assessment of the included studies.
- Only studies published in English will be included.

# Introduction

## Background

Eating disorders (EDs) are complex psychiatric conditions characterised by disordered eating and/or weight-control behaviours [1] which typically have their onset during adolescence [2]. A recent systematic review reported a pooled global lifetime and 12-month prevalence of EDs of 0.91% and 0.43%, respectively [3]. EDs have profound physical and psychological impacts on the affected individual and those around them. EDs have high co-morbidity with other psychiatric conditions and are associated with suicidal thoughts and attempts [4,5]. The medical complications that often accompany EDs involve almost all organ systems and can be life-threatening [6], leading to significantly increased mortality rates [7]. Given the negative consequences of EDs, it is imperative that we understand their prevalence and treatment within and across ethnic groups.

Despite the prevailing stereotype that EDs only affect "skinny, White, affluent girls" (the SWAG stereotype) [8], it is widely reported that EDs affect individuals from all racial and ethnic backgrounds [9]. However, findings regarding the prevalence of disordered eating and EDs across ethnic groups are mixed. Some studies report no difference in disordered eating and ED prevalence across different ethnic groups [10-12]. Others, however, have reported higher prevalence of disordered eating among minority ethnic men and women relative to White individuals [13]. In contrast, some studies have found higher prevalence of EDs in White adults when compared to other racial and ethnic groups [8,14,15]. Reported prevalence seems to vary based on the racial or ethnic group and type of disordered eating or ED being studied [9]. Differences in study design and diagnostic criteria used may also contribute to differences in reported prevalence [11].

Despite similar and in some cases increased rates of disordered eating and EDs among minority ethnic groups there appear to be disparities when it comes to ED diagnosis and treatment. In their systematic review, Sinha and Warfa [16] found minority ethnic individuals in the UK and US were less likely to seek and receive ED treatment as well as less frequently diagnosed and referred to specialist ED services. Since publication of this review further studies have replicated these findings [8,17,18]. Such disparities in diagnosis and treatment have been attributed to a lack of perceived need for treatment



For peer review only - http://bmjopen.bmj.com/site/about/guidelines.xhtml

For peer review only

among these populations [8], differences in symptom presentation [18] and culturally insensitive diagnostic criteria [19].

To date, most of the existing literature exploring disordered eating and EDs among minority ethnic groups has been conducted in the United States (US). This is apparent in recent narrative and systematic reviews which have explored the identification, prevalence, presentation, risk factors and treatment of disordered eating and EDs among minority racial and ethnic populations [9,16,20-23]. The overwhelming majority of studies included in these reviews have been conducted in the US.

Researchers have called for more research exploring ethnic and racial diversity in EDs to be conducted outside of the US [24]. Generalisability of findings from US studies to countries with different healthcare systems and sociocultural and environmental contexts has been questioned [11,25]. Franko and Rodgers [26] also argue research outside of the US is necessary to fully understand the extent of similarities and differences within and across ethnic groups.

## Rationale

The present scoping review aims to identify and describe studies reporting on disordered eating and EDs in minority ethnic groups in the UK, Canada, Australia and New Zealand. These countries have large established minority ethnic populations due to high levels of immigration and/or the forced displacement of Indigenous peoples, as well as broadly comparable healthcare systems. By providing a broad overview of the extent, nature and range of existing research conducted in these countries we hope to elucidate any knowledge gaps to inform future research.

Given the well-documented racial and ethnic health disparities in the diagnosis and treatment of EDs [16] and mental health conditions more broadly [27], a special focus will be placed on studies which have reported on the prevalence of disordered eating and EDs, access to care and treatment experiences among minority ethnic groups. Such studies will be particularly relevant in identifying any health disparities in the countries of focus and highlighting possible ways to overcome these. This is of particular importance given prompt diagnosis and intervention can improve ED treatment outcomes and recovery prospects [28].

## Objectives

In this scoping review we aim to examine the extent, range and nature of research activity into disordered eating and EDs among minority ethnic populations in the UK, Canada, Australia and New Zealand. The specific objectives are to:



For peer review only - http://bmjopen.bmj.com/site/about/guidelines.xhtml

1. Map the literature according to the main themes/topics addressed, target populations and methodologies utilised;
2. Report on findings regarding ED prevalence, access to care and treatment experiences;
3. Identify gaps and/or limitations in the literature and suggest areas for future research.

# Methods and Analysis

## Protocol Design

A scoping review was chosen as the most appropriate methodology. Scoping reviews are suited to answering broad research questions and are designed to identify the types of evidence available in a field, examine how this research has been conducted and highlight any knowledge gaps [29]. The present scoping review will follow the five stage framework first outlined by Arksey and O'Malley [30] and subsequently improved by Levac et al [31]. The stages are as follows: (1) identifying the research questions; (2) identifying the relevant literature; (3) selecting studies; (4) charting the data; and (5) collating, summarising and reporting the results. To ensure quality of the scoping review, the development and reporting of this protocol followed the Preferred Reporting Items for Systematic reviews and Meta-Analyses Extension for Scoping Reviews (PRISMA-ScR) guidelines [32]. The protocol for this scoping review is also registered on OSF (https://osf.io/w3d6g). Any changes made as part of the iterative nature of the scoping review will be documented here.

## Stage 1: Identifying the Research Questions

The first stage of a scoping review is to develop research questions informed by the purpose and objectives of the scoping review[31]. Based on the aforementioned objectives of this scoping review we have formulated six research questions. These are described in further detail below. Given the iterative nature of scoping reviews, a reflexive approach will be adopted. If necessary, as we become more familiar with the existing literature our research questions will be refined and modified accordingly.

**Question 1: What topics are being addressed by the current literature?**

Within the US studies have explored the identification, prevalence, presentation, risk factors and treatment of EDs among minority racial and ethnic groups. For each study included in this scoping review we will report on the primary focus of the study. By doing so we hope to provide a summary of the themes/topics that have been addressed by the current literature on disordered eating and EDs among minority ethnic populations in the UK, Canada, Australia and New Zealand.



For peer review only - http://bmjopen.bmj.com/site/about/guidelines.xhtml

**Question 2: How has this research been conducted?**

This research question aims to explore how the included studies have been designed and conducted. For each study we will report the research design and methodology used to answer research questions.

**Question 3: Which populations have been targeted for this research?**

By answering this question we hope to provide an overview of which groups and populations have been studied. We will report on key participant characteristics (e.g., age, gender, socioeconomic status, co-morbidities) and examine which ethnic groups and types of disordered eating or EDs have been studied. We also aim to explore how and where participants were recruited (e.g., sampling technique, clinical or non-clinical sample, sample size).

**Question 4: Are there differences in reported ED prevalence across ethnicity?**

This question aims to assess whether reported prevalence rates of disordered eating and EDs differ across ethnic groups.

**Question 5: Are there differences in access to ED treatment across ethnic groups?**

To answer this question we will extract and analyse data from studies which have reported findings regarding any of the following: referrals to ED services, service utilisation, barriers to care or help-seeking (or treatment-seeking).

**Question 6: Do ED treatment experiences differ across ethnic groups?**

The purpose of this question is to assess what is currently known regarding the treatment experiences of minority ethnic patients. We will aim to explore how minority ethnic individuals and/or stakeholders involved in the delivery or receipt of their treatment (e.g., clinicians, family members) describe their experiences of treatment. We will also aim to identify the types of treatment that have been used in minority ethnic populations.

## Stage 2: Identifying Relevant Studies

**Information Sources**

The second stage of a scoping review is to identify relevant studies for selection and data extraction. This will involve a systematic search of the following electronic databases: (1) Medline (2) PsycINFO; (3) EMBASE; (4) CINAHL and (5) Web of Science. To ensure the search is as comprehensive as possible, backwards citation chaining of included studies will be used to identify any additional studies of relevance. For sake of completeness, a web search using Google Scholar will also be completed. Both published and grey literature (e.g., theses) will be considered for inclusion.



For peer review only - http://bmjopen.bmj.com/site/about/guidelines.xhtml

For peer review only

**Search Strategy**

The search strategy was developed by members of the research team (HT, SM, and RM) in collaboration with a specialty academic support librarian. Search terms include keywords and terms related to (1) EDs and (2) ethnicity. See online supplement appendix 1 for the search strategy for each of the databases. No date limits will be applied to the search results, but the database search query will be set to produce articles in English only due to resource constraints.

The search results for each database will be documented and references will be imported into the citation management system Endnote, where duplicates will be removed before studies are screened for inclusion.

## Stage 3: Selecting Studies

The process of selecting studies will consist of two stages: (1) title and abstract screening and (2) full-text screening. Each stage will involve two data reviewers independently screening the identified studies against the inclusion and exclusion criteria. All screening will be conducted using the online screening tool Rayyan.

In the first round of screening two reviewers (RM and HT) will review paper titles and abstracts. Papers will be categorised as either 'include', 'exclude' or 'maybe'. Decisions as to whether papers are eligible for full-text review will be made as follows: (1) if both reviewers agree to 'include', the study will move on to the second screening; (2) if both reviewers agree to 'exclude', the study will not be read in full and will be excluded from the review; (3) if the reviewers are not unanimous in their agreement, the study will also move onto the second screening to be read in full before a final decision is made. Where a paper abstract is unavailable, papers will be assigned to the 'maybe' category and automatically move on to the second stage of screening to be read in full. Eligibility criteria will be further refined and finalised following the first round of screening if necessary.

In the second round of screening two reviewers (AWR and HT) will read the remaining articles in full to determine whether they meet the inclusion and exclusion criteria. If the decision to include or exclude if not unanimous, both reviewers will assess the study together and discuss disagreements. A third reviewer may be consulted for arbitration of any conflicting decisions. A flowchart outlining decisions made regarding study inclusion will be produced using the PRIMSA template [33].

**Eligibility Criteria**

Eligibility criteria were developed using the population, context, concept (PCC) framework [34] (see table 1). Inclusion and exclusion criteria are further detailed below:



For peer review only - http://bmjopen.bmj.com/site/about/guidelines.xhtml

**Table 1**

Core elements of the scoping review based on Population Concept, Context (PCC) Framework

| | | |
|---|---|---|
| **Population** | Minority Ethnic Groups | A group of people within a country that: "is numerically smaller than the rest of the population; is not in a dominant position; has a culture, language, religion or race that is distinct from that of the majority; and its members have a will to preserve those characteristics" [35 as cited in 36, p.97] |
| **Concept** | Eating Disorders | Clinical psychiatric disorders, including anorexia nervosa, bulimia nervosa and binge eating disorder, characterised by disturbed eating behaviours, body image concerns and unhealthy weight control behaviours[1]. |
| | Disordered Eating | Subclinical levels of unhealthy eating behaviours including restrictive eating, binge eating, unhealthy weight control behaviours [37. |
| **Context** | Studies conducted in the UK, Canada, Australia or New Zealand. | |



For peer review only - http://bmjopen.bmj.com/site/about/guidelines.xhtml

**Table 2**

Inclusion and Exclusion Criteria

| | Inclusion | Exclusion |
|---|---|---|
| Population | - Sub- or full sample of minority ethnic participants or data related to minority ethnic individuals<br>- Where multiple ethnic groups are included in a sample the findings are reported according to ethnicity. For example, ethnicity is the main predictor variable in analyses or the paper reports on ethnic differences in study outcomes (e.g., prevalence rates, scores on psychometric measures, referral rates etc.).<br>- All ages | - Where multiple minority ethnic groups are included in the sample, but authors did not report any outcomes or analysis according to ethnicity (e.g., ethnicity only reported in sample demographics, analyses reported for sample as a whole rather than individual ethnic groups, where analyses are controlled or adjusted for ethnicity but no further findings or data relating to ethnicity were reported). |
| Concept | - Study focuses on clinical EDs (i.e., meeting DSM or ICD criteria as assessed using clinician interview or a validated psychometric measure) or disordered eating (i.e., assessed with a validated psychometric measure). | - Focuses solely on risk factors, associated concepts or related conditions (e.g., food addiction, obesity, body image).<br>- Focuses solely on ARFID, pica or rumination disorder. |
| Context | - Study conducted in UK, Canada, Australia or New Zealand. | |
| Study Design | - Any study design (e.g, case reports, case studies, cohort studies, randomized controlled trials etc.) | |



For peer review only - http://bmjopen.bmj.com/site/about/guidelines.xhtml

| | | |
|---|---|---|
| Publication Type | - Peer reviewed journal articles or theses | - Review articles, editorials, commentaries, letters, newsletters, opinion or reflection pieces, book chapters, book reviews, poster presentations or conference abstracts. |
| Language | - Published in English | |

## Stage 4: Charting the Data/Data Extraction

Once studies have been identified for final inclusion, data from these studies will be extracted and charted. A comprehensive data charting form will be developed in Excel and used to extract relevant information from studies. Two reviewers (AWR and HT) will pilot the data charting form on a sample of randomly selected papers (10% of included papers). Any discrepancies or uncertainties highlighted by this exercise will be discussed and the charting form will be adjusted accordingly. One reviewer (AWR) will extract the data from the remaining papers. The research team will meet regularly to discuss progress throughout the data extraction process and further changes to the data extraction form will be made as needed in an iterative process. For example, additional categories of interest may emerge. Any changes made and the rationale for doing so will be reported in final scoping review findings.

Data to be extracted will include some standard information about the paper. For example, study title, author(s), year of publication and country of publication. Additional information specific to our research questions will also be extracted. This will include the study aims/objectives, research focus/topic, study design and methodology, sample characteristics and demographics (e.g., age, gender, ED diagnosis, ethnicity, sample size), setting in which the study was conducted (e.g., clinical or non-clinical setting), summary of relevant findings, and recommendations of the author(s) if applicable. In line with guidance on conducting scoping review, there will be no critical appraisal of the included studies [32].

## Stage 5: Collating, Summarising and Reporting the Results

Rather than meta-synthesis or critical appraisal of studies, the purpose of a scoping review is to provide an overview of the existing literature, summarising what is already known and identifying any knowledge gaps [29]. The extracted data will be summarised in a way that maps the extent, range and



For peer review only - http://bmjopen.bmj.com/site/about/guidelines.xhtml

nature of research into disordered eating and EDs among minority ethnic populations in the UK, Canada, Australia and New Zealand, whilst placing particular focus on differences in prevalence, access to care and treatment experiences across ethnicity.

We will provide a descriptive numerical summary of the characteristics of included studies. Studies will be classified and grouped according to their characteristics. Extracted data will be presented in tables, charts and visual maps. To accompany tabular and visual summaries, we will also provide a qualitative narrative summary of the data and how it relates to our research questions. If data and resources permit, thematic analysis will also be completed. We will report on themes relevant to our research questions (e.g., barriers to accessing care, descriptions of treatment experience), as well as any other themes which may arise.

Finally, informed by any significant gaps and/or limitations of the literature we will recommend avenues for future research. The scoping review findings will also be discussed in relation to clinical practice and policy. Reporting of the results will follow PRISMA ScR guidelines[32].

## Ethics and Dissemination

A preliminary search of MEDLINE, PROSPERO, the Cochrane Database of Systematic Reviews and *JBI Evidence Synthesis* was conducted and no current or underway systematic reviews or scoping reviews on the present topic were identified. To our knowledge, this will be the first systematic scoping review of the literature exploring disordered eating and EDs in minority ethnic populations in the UK, Canada, Australia and New Zealand.

This study will provide a comprehensive and high-quality summary of the available literature. Through identification of knowledge gaps, this scoping review may be used to inform the direction of future research. This is of particular importance given the identified need for more research exploring racial and ethnic differences in EDs outside of the US [25]. Our research questions related to ethnic differences in prevalence, access to care and treatment experiences and outcomes specifically may also be of interest to researchers, clinicians and policy makers. For example, by identifying possible improvements to service structure and provision which may improve the accessibility and effectiveness of ED treatment. As scoping reviews involve the methodological examination and integration of existing literature and resources ethical approval is not required.

Once all stages of the scoping review are complete, findings will be disseminated via conferences, presentations and the submission of the final review for peer-reviewed publication.



For peer review only - http://bmjopen.bmj.com/site/about/guidelines.xhtml

## Patient and Public Involvement

Patients or the public have not been involved in the design, conduct, reporting, or dissemination plans of this scoping review.

## Acknowledgements

The authors thank Samantha Johnson, Academic Support Librarian, for assisting in the search strategy.

## Author Contributions

HT and SM were involved in the initial conception of the review. HT is responsible for the design of the review. RM aided with the design of the review and drafted the first manuscript. AWR revised the manuscript making critical edits. KG contributed meaningfully to the drafting and editing of the manuscript. All authors read and approved the final manuscript.

## Funding Statement

This research is funded by the National Institute for Health and Care Research (NIHR) Applied Research Collaboration (ARC) West Midlands. The views expressed are those of the authors and not necessarily those of the NIHR or the Department of Health and Social Care.

## Competing Interests Statement

None declared.

## Word Count

2606

For peer review only

# References


1. American Psychiatric Association. Diagnostic and Statistical Manual of Mental Sisorders (5th edn.) APA, 2013.

2. Volpe U, Tortorella A, Manchia M, et al. Eating disorders: What age at onset? Psychiatry Res. 2016;238:225-7.

3. Qian J, Wu Y, Liu F, et al. An update on the prevalence of eating disorders in the general population: a systematic review and meta-analysis. Eating and Weight Disorders-Studies on Anorexia, Bulimia and Obesity. 2021:1-14.

4. Chamay-Weber C, Narring F, Michaud PA. Partial eating disorders among adolescents: A review. J of Adolesc Health. 2005;37(5):416-26.

5. Lewinsohn PM, Striegel-Moore RH, Seeley JR. Epidemiology and natural course of eating disorders in young women from adolescence to young adulthood. J Am Academy of Child Adolesc Psychiatry. 2000;39(10):1284-92.

6. Voderholzer U, Haas V, Correll CU, et al. Medical management of eating disorders: an update. Curr Opin Psychiatry. 2020;33(6):542-53.

7. van Hoeken D, Hoek HW. Review of the burden of eating disorders: mortality, disability, costs, quality of life, and family burden. Curr Opin Psychiatry. 2020;33(6):521-27.

8. Sonneville K, Lipson S. Disparities in eating disorder diagnosis and treatment according to weight status, race/ethnicity, socioeconomic background, and sex among college students. Int J Eat Disord. 2018;51(6):518-26.

9. Rodgers RF, Berry R, Franko DL. Eating disorders in ethnic minorities: An update. Curr Psychiatry Rep. 2018;20:1-11.

10. Cheng ZH, Perko VL, Fuller-Marashi L, et al. Ethnic differences in eating disorder prevalence, risk factors, and predictive effects of risk factors among young women. Eat Behav. 2019;32:23-30.


For peer review only



For peer review only - http://bmjopen.bmj.com/site/about/guidelines.xhtml

11. Solmi F, Hotopf M, Hatch S, et al. Eating disorders in a multi-ethnic inner-city UK sample: prevalence, comorbidity and service use. Soc Psychiatry Psychiatr Epidemiol. 2016;51:369-81.

12. Marques L, Alegria M, Becker AE, et al. Comparative prevalence, correlates of impairment, and service utilization for eating disorders across US ethnic groups: Implications for reducing ethnic disparities in health care access for eating disorders. Int J Eat Disord. 2011;44(5):412-20.

13. Simone M, Telke S, Anderson LM, et al. Ethnic/racial and gender differences in disordered eating behavior prevalence trajectories among women and men from adolescence into adulthood. Soc Sci Med. 2022;294:114720.

14. Udo T, Grilo CM. Prevalence and correlates of DSM-5–defined eating disorders in a nationally representative sample of US adults. Biol Psychiatry. 2018;84(5):345-54.

15. Striegel-Moore RH, Dohm FA, Kraemer HC, et al. Eating disorders in white and black women. Am J Psychiatry. 2003;160(7):1326-31.

16. Sinha S, Warfa N. Treatment of eating disorders among ethnic minorities in western settings: a systematic review. Psychiatr Danub. 2013;25(2):295-9.

17. Coffino JA, Udo T, Grilo CM. Rates of help-seeking in US adults with lifetime DSM-5 eating disorders: prevalence across diagnoses and differences by sex and ethnicity/race. Mayo Clin Proc. 2019;94(8):1415-1426.

18. Solmi F, Hatch SL, Hotopf M, et al. Prevalence and correlates of disordered eating in a general population sample: the South East London Community Health (SELCoH) study. Soc Psychiatry Psychiatr Epidemiol. 2014;49:1335-46.

19. Lee-Winn A, Mendelson T, Mojtabai R. Racial/ethnic disparities in binge eating: disorder prevalence, symptom presentation, and help-seeking among Asian Americans and non-Latino Whites. Am J Public Health. 2014;104(7):1263-5.

20. Goode RW, Cowell MM, Mazzeo SE, et al. Binge eating and binge-eating disorder in Black women: A systematic review. Int J Eat Disord. 2020;53(4):491-507.

For peer review only

21. Acle A, Cook BJ, Siegfried N, et al. Cultural considerations in the treatment of eating disorders among racial/ethnic minorities: A systematic review. J Cross Cult Psychol. 2021;52(5):468-88.

22. Song S, Stern CM, Deitsch T, et al. Acculturation and eating disorders: a systematic review. Eating and Weight Disorders-Studies on Anorexia, Bulimia and Obesity. 2023;28(1):39.

23. Doris E, Shekriladze I, Javakhishvili N, et al. Is cultural change associated with eating disorders? A systematic review of the literature. Eating and Weight Disorders-Studies on Anorexia, Bulimia and Obesity. 2015;20:149-60.

24. Egbert AH, Hunt RA, et al. Reporting racial and ethnic diversity in eating disorder research over the past 20 years. Int J Eat Disord. 2022;55(4):455-62.

25. Mikhail ME, Klump KL. A virtual issue highlighting eating disorders in people of black/African and Indigenous heritage. Int J Eat Disord. 2021;54(3):459-67.

26. Franko DL, Rodgers RF. Ethnicity and eating disorders: More similarities than differences. Encyclopedia of Feeding and Eating Disorders. 2017;366-70.

27. Maguire TG, Miranda J. Racial and ethnic disparities in mental healthcare: evidence and policy implications. Health Aff. 2008;27:393-403.

28. Linardon J, Messer M, Rodgers RF, et al. A systematic scoping review of research on COVID-19 impacts on eating disorders: A critical appraisal of the evidence and recommendations for the field. Int J Eat Disord. 2022;55(1):3-8.

29. Munn Z, Peters MD, Stern C, et al. Systematic review or scoping review? Guidance for authors when choosing between a systematic or scoping review approach. BMC Med Res Methodol. 2018;18:1-7.

30. Arksey H, O'Malley L. Scoping studies: towards a methodological framework. Int J Soc Res Methodol. 2005;8(1):19-32.

31. Levac D, Colquhoun H, O'Brien KK. Scoping studies: advancing the methodology. Implementation Sci. 2010;5:1-9.



For peer review only - http://bmjopen.bmj.com/site/about/guidelines.xhtml

32. Tricco AC, Lillie E, Zarin W, et al. PRISMA extension for scoping reviews (PRISMA-ScR): checklist and explanation. Ann Internal Med. 2018;169(7):467-73.

33. Moher D, Liberati A, Tetzlaff J, et al. Preferred reporting items for systematic reviews and meta-analyses: the PRISMA statement. Ann Internal Med. 2009;151(4):264-9.

34. Aromataris E, Munn Z, eds. JBI manual for evidence synthesis [e-book]. JBI; 2020. https://synthesismanual.jbi.global [accessed 11 Dec 2023].

35. Foa, RS. Creating an Inclusive Society: Evidence from Social Indicators and Trends. [online]. 2015. https://www.repository.cam.ac.uk/handle/1810/342924 [accessed 11 Dec 2023].

36. United Nations. Indigenous Peoples and Ethnic Minorities: Marginalization Is the Norm. In: The Report on the World Social Situation. 2018:97-108.

37. Krentz EM, Warschburger P. Sports-related correlates of disordered eating in aesthetic sports. Psychol of Sport Exerc. 2011;12(4):375-82.

For peer review only

Database Searches

**Medline**

Ovid MEDLINE(R) ALL <1946 to March 30, 2023>

1 "feeding and eating disorders"/ or anorexia nervosa/ or avoidant restrictive food intake disorder/ or binge-eating disorder/ or bulimia nervosa/ or diabulimia/ or "feeding and eating disorders of childhood"/ or food addiction/ or night eating syndrome/ or orthorexia nervosa/ or pica/ or relative energy deficiency in sport/ or female athlete triad syndrome/ or rumination syndrome/ 35804

2 eating disorder*.mp. 33463

3 anorexi*.mp. 42181

4 bulimi*.mp. 12324

5 ARFID.mp. 268

6 avoidant restrictive food intake disorder.mp. 432

7 binge eating.mp. 7407

8 pica.mp. 3495

9 rumination disorder.mp. 41

10 rumination syndrome.mp. 162

11 1 or 2 or 3 or 4 or 5 or 6 or 7 or 8 or 9 or 10 73164

12 population groups/ or african people/ or north african people/ or sub-saharan african people/ or central african people/ or east african people/ or southern african people/ or west african people/ or asian people/ or asian/ or central asian people/ or east asian people/ or north asian people/ or southeast asian people/ or west asian people/ or middle eastern people/ or south asian people/ or black people/ or "black or african american"/ or caribbean people/ or central american people/ or indians, central american/ or european people/ or eastern european people/ or "scandinavians and nordic people"/ or "middle eastern and north africans"/ or north american people/ or "american indian or alaska native"/ or indians, north american/ or alaskan natives/ or indigenous canadians/ or inuit/ or navajo people/ or pima people/ or population groups, us/ or ethnicity/ or "hispanic or latino"/ or mexican americans/ or racial groups/ or "native hawaiian or other pacific islander"/ or white/ or oceanians/ or australasian people/ or "australian aboriginal and torres strait islander peoples"/ or pacific island people/ or maori people/ or south american people/ or indians, south american/ or white people/ 326840

13 "emigrants and immigrants"/ or undocumented immigrants/ or refugees/ or "transients and migrants"/ 39912

14 "black or african american"/ or amish/ or arabs/ or "asian american native hawaiian and pacific islander"/ or asian/ or "native hawaiian or other pacific islander"/ or "hispanic or latino"/ or mexican americans/ or indigenous peoples/ or "american indian or alaska native"/ or indians, central american/ or indians, north american/ or alaskan natives/ or indigenous canadians/ or inuit/ or

For peer review only

navajo people/ or pima people/ or indians, south american/ or "australian aboriginal and torres strait islander peoples"/ or maori people/ or roma/ 133629

15 asia, southern/ or afghanistan/ or bangladesh/ or bhutan/ or india/ or sikkim/ or maldives/ or nepal/ or pakistan/ or sri lanka/ 172233

16 ethnic minorit*.mp. 15411

17 racial minorit*.mp. 2337

18 multi-ethnic*.mp. 5370

19 "ethnic and racial minorities"/ or minority groups/ 17800

20 BAME.mp. 358

21 BIPOC.mp. 264

22 people of colo*r.mp. 1373

23 12 or 13 or 14 or 15 or 16 or 17 or 18 or 19 or 20 or 21 or 22 549059

24 11 and 23 1312

For peer review only

**EMBASE**

Embase Classic+Embase <1947 to 2023 Week 12>

1 eating disorder/ or anorexia nervosa/ or avoidant restrictive food intake disorder/ or binge eating disorder/ or bulimia/ or emotional eating/ or food addiction/ or food aversion/ or food neophobia/ or food refusal/ or muscle dysmorphia/ or orthorexia/ or pica/ or purging disorder/ or relative energy deficiency in sport/ 64869

2 (feeding and eating disorders).mp. [mp=title, abstract, heading word, drug trade name, original title, device manufacturer, drug manufacturer, device trade name, keyword heading word, floating subheading word, candidate term word]5597

3 diabulimia.mp. 64

4 (feeding and eating disorders of childhood).mp. [mp=title, abstract, heading word, drug trade name, original title, device manufacturer, drug manufacturer, device trade name, keyword heading word, floating subheading word, candidate term word] 52

5 night eating syndrome.mp. 465

6 rumination syndrome/ or feeding disorder/ 6564

7 eating disorder*.mp. [mp=title, abstract, heading word, drug trade name, original title, device manufacturer, drug manufacturer, device trade name, keyword heading word, floating subheading word, candidate term word]47823

8 anorexi*.mp. [mp=title, abstract, heading word, drug trade name, original title, device manufacturer, drug manufacturer, device trade name, keyword heading word, floating subheading word, candidate term word] 115739

9 bulimi*.mp. [mp=title, abstract, heading word, drug trade name, original title, device manufacturer, drug manufacturer, device trade name, keyword heading word, floating subheading word, candidate term word] 18145

10 ARFID.mp. [mp=title, abstract, heading word, drug trade name, original title, device manufacturer, drug manufacturer, device trade name, keyword heading word, floating subheading word, candidate term word] 438

11 avoidant restrictive food intake disorder.mp. [mp=title, abstract, heading word, drug trade name, original title, device manufacturer, drug manufacturer, device trade name, keyword heading word, floating subheading word, candidate term word] 698

12 binge eating.mp. [mp=title, abstract, heading word, drug trade name, original title, device manufacturer, drug manufacturer, device trade name, keyword heading word, floating subheading word, candidate term word] 12511

13 pica.mp. [mp=title, abstract, heading word, drug trade name, original title, device manufacturer, drug manufacturer, device trade name, keyword heading word, floating subheading word, candidate term word] 4755

For peer review only

14 rumination disorder.mp. [mp=title, abstract, heading word, drug trade name, original title, device manufacturer, drug manufacturer, device trade name, keyword heading word, floating subheading word, candidate term word]57

15 rumination syndrome.mp. [mp=title, abstract, heading word, drug trade name, original title, device manufacturer, drug manufacturer, device trade name, keyword heading word, floating subheading word, candidate term word]414

16 1 or 2 or 3 or 4 or 5 or 6 or 7 or 8 or 9 or 10 or 11 or 12 or 13 or 14 or 15 165879

17 population group/ or ancestry group/ or citizen group/ or ethnic group/ or minority group/ 107708

18 ancestry group/ or asian american/ or asian continental ancestry group/ or australoid/ or black person/ or british asian/ or caucasian/ or hispanic/ or indigenous people/ or migrant/ or mongoloid/ or multiracial person/ or oceanic ancestry group/ 279452

19 black person/ or african american/ or african brazilian/ or african caribbean/ 144776

20 caucasian/ or european american/ 172363

21 hispanic/ or mexican american/ 91634

22 indigenous people/ or alaska native/ or american indian/ or canadian aboriginal/ or first nation/ or indigenous australian/ or taiwanese aborigine/ 35946

23 migrant/ or emigrant/ or forced migrant/ or immigrant/ or migrant worker/ 34289

24 multiracial person/ 711

25 oceanic ancestry group/ or pacific islander/ or torres strait islander/ 10630

26 citizen group/ or african/ or asian/ or "caribbean (person)"/ or central american/ or european/ or north american/ or oceanian/ or south american/ 255326

27 african/ or central african/ or east african/ or north african/ or southern african/ or west african/ 11807

28 central african/ or angolan/ or cameroonian/ or "chadian (citizen)"/ or "citizen of the central african republic"/ or "congolese (brazzaville)"/ or "congolese (kinshasa)"/ or gabonese/ 855

29 east african/ or eritrean/ or ethiopian/ or kenyan/ or "malagasy (citizen)"/ or malawian/ or mauritian/ or mozambican/ or rwandan/ or "somali (citizen)"/ or tanzanian/ or ugandan/ or zambian/ or zimbabwean/ 6458

30 north african/ or algerian/ or egyptian/ or libyan/ or moroccan/ or sudanese/ or tunisian/ 11694

31 southern african/ or namibian/ or south african/ or "swazi (citizen)"/ 4278

32 west african/ or beninese/ or burkinabe/ or gambian/ or ghanaian/ or guinean/ or "ivorian (citizen)"/ or liberian/ or malian/ or mauritanian/ or nigerian/ or nigerien/ or senegalese/ or sierra leonean/ or togolese/ 5473

33 asian/ or central asian/ or east asian/ or south asian/ or southeast asian/ or west asian/ 106199

For peer review only

34 central asian/ or kazakhstani/ or "kyrgyz (citizen)"/ or "tajik (citizen)"/ or "turkmen (citizen)"/ or "uzbek (citizen)"/ 405

35 east asian/ or chinese/ or "japanese (citizen)"/ or "mongolian (citizen)"/ or north korean/ or south korean/ or taiwanese/ 93841

36 south asian/ or afghan/ or bangladeshi/ or bhutanese/ or indian/ or nepalese/ or pakistani/ or sri lankan/ 51986

37 southeast asian/ or burmese/ or cambodian/ or "filipino (citizen)"/ or indonesian/ or laotian/ or malaysian/ or singaporean/ or "thai (citizen)"/ or vietnamese/11375

38 west asian/ or "armenian (citizen)"/ or azerbaijani/ or bahraini/ or cypriot/ or emirati/ or "georgian (citizen)"/ or "iranian (citizen)"/ or iraqi/ or israeli/ or jordanian/ or kuwaiti/ or lebanese/ or omani/ or palestinian/ or qatari/ or saudi/ or syrian/ or turkish citizen/ or yemeni/ 16521

39 caribbean/ or antillean/ 5271

40 central american/ or belizean/ or costa rican/ or guatemalan/ or honduran/ or mexican/ or nicaraguan/ or panamanian/ or salvadoran/ 6159

41 european/ or central european/ or eastern european/ or eu citizen/ or northern european/ or southern european/ or western european/ 146844

42 central european/ or austrian/ or "czech (citizen)"/ or "german (citizen)"/ or "hungarian (citizen)"/ or polish citizen/ or "slovak (citizen)"/ or "slovenian (citizen)"/ or swiss/ 18342

43 eastern european/ or "armenian (citizen)"/ or azerbaijani/ or "belarusian (citizen)"/ or "bulgarian (citizen)"/ or "czech (citizen)"/ or "georgian (citizen)"/ or "hungarian (citizen)"/ or polish citizen/ or "romanian (citizen)"/ or "russian (citizen)"/ or "slovak (citizen)"/ or "ukrainian (citizen)"/ 9113

44 eu citizen/ or austrian/ or belgian/ or "bulgarian (citizen)"/ or "croatian (citizen)"/ or cypriot/ or "czech (citizen)"/ or danish citizen/ or dutchman/ or "estonian (citizen)"/ or "finn (citizen)"/ or frenchman/ or "german (citizen)"/ or "greek (citizen)"/ or "hungarian (citizen)"/ or "irish (citizen)"/ or "italian (citizen)"/ or "latvian (citizen)"/ or "lithuanian (citizen)"/ or "maltese (citizen)"/ or polish citizen/ or "portuguese (citizen)"/ or "romanian (citizen)"/ or "slovak (citizen)"/ or "slovenian (citizen)"/ or spaniard/ or swedish citizen/ 52321

45 northern european/ or british citizen/ or danish citizen/ or "estonian (citizen)"/ or faroese/ or "finn (citizen)"/ or icelander/ or "irish (citizen)"/ or "latvian (citizen)"/ or "lithuanian (citizen)"/ or "norwegian (citizen)"/ or swedish citizen/ 14870

46 british citizen/ or briton/ or northern irish/ 1731

47 briton/ or englishman/ or scotsman/ or welshman/ 1166

48 southern european/ or "albanian (citizen)"/ or "bosnian (citizen)"/ or "croatian (citizen)"/ or cypriot/ or "greek (citizen)"/ or "italian (citizen)"/ or kosovar/ or "macedonian (citizen)"/ or "maltese (citizen)"/ or "portuguese (citizen)"/ or "serbian (citizen)"/ or "slovenian (citizen)"/ or spaniard/ or yugoslav/ 18026

49 western european/ or austrian/ or belgian/ or dutchman/ or frenchman/ or "german (citizen)"/ or swiss/ 22080

For peer review only

50 north american/ or american/ or canadian/ 173924

51 oceanian/ or australian/ or melanesian/ or micronesian/ or new zealander/ or polynesian/ 16781

52 melanesian/ or fijian/ or new caledonian/ or papua new guinean/ 276

53 micronesian/ or marshallese/ or nauruan/ or palauan/ 94

54 polynesian/ or american samoan/ or cook islander/ or french polynesian/ or "hawaiian (citizen)"/ or tongan/ or western samoan/ 406

55 south american/ or argentinian/ or bolivian/ or brazilian/ or chilean/ or colombian/ or ecuadorean/ or guianese/ or guyanese/ or paraguayan/ or peruvian/ or surinamese/ or uruguayan/ or venezuelan/ 17847

56 ethnic group/ or afro-asiatic people/ or altaic people/ or amerind people/ or amish/ or australian aborigine/ or austroasiatic people/ or austronesian people/ or "basque (people)"/ or caucasian speaking people/ or dravidian people/ or eskimo-aleut people/ or "hadza (people)"/ or hmong-mien people/ or indo-european people/ or khoisan/ or na-dene people/ or negrito/ or niger-congo people/ or nilo-saharan people/ or paleosiberian people/ or papuan people/ or pygmy/ or sino-tibetan people/ or tai-kadai people/ or uralic people/ 85845

57 ethnic group/ or exp afro-asiatic people/ or exp altaic people/ or exp amerind people/ or exp amish/ or exp australian aborigine/ or exp austroasiatic people/ or exp austronesian people/ or exp "basque (people)"/ or exp caucasian speaking people/ or exp dravidian people/ or exp eskimo-aleut people/ or exp "hadza (people)"/ or exp hmong-mien people/ or exp indo-european people/ or exp khoisan/ or exp na-dene people/ or exp negrito/ or exp niger-congo people/ or exp nilo-saharan people/ or exp paleosiberian people/ or exp papuan people/ or exp pygmy/ or exp sino-tibetan people/ or exp tai-kadai people/ or exp uralic people/ 190016

58 ethnic minorit*.mp. [mp=title, abstract, heading word, drug trade name, original title, device manufacturer, drug manufacturer, device trade name, keyword heading word, floating subheading word, candidate term word] 20056

59 racial minorit*.mp. [mp=title, abstract, heading word, drug trade name, original title, device manufacturer, drug manufacturer, device trade name, keyword heading word, floating subheading word, candidate term word] 2662

60 multi-ethnic*.mp. [mp=title, abstract, heading word, drug trade name, original title, device manufacturer, drug manufacturer, device trade name, keyword heading word, floating subheading word, candidate term word] 9364

61 BAME.mp. [mp=title, abstract, heading word, drug trade name, original title, device manufacturer, drug manufacturer, device trade name, keyword heading word, floating subheading word, candidate term word] 595

62 BIPOC.mp. [mp=title, abstract, heading word, drug trade name, original title, device manufacturer, drug manufacturer, device trade name, keyword heading word, floating subheading word, candidate term word] 332

63 17 or 18 or 19 or 20 or 21 or 22 or 23 or 24 or 25 or 26 or 27 or 28 or 29 or 30 or 31 or 32 or 33 or 34 or 35 or 36 or 37 or 38 or 39 or 40 or 41 or 42 or 43 or 44 or 45 or 46 or 47 or 48 or 49 or 50 or 51 or 52 or 53 or 54 or 55 or 56 or 57 or 58 or 59 or 60 or 61 or 62 1199105

For peer review only

| | | |
|---|---|---|
| 64 | 16 and 63 | 6820 |

For peer review only

**PsycInfo**

APA PsycInfo <1806 to March Week 3 2023>

1 eating disorders/ or anorexia nervosa/ or "avoidant/restrictive food intake disorder"/ or binge eating disorder/ or bulimia/ or feeding disorders/ or hyperphagia/ or kleine levin syndrome/ or orthorexia/ or pica/ or "purging (eating disorders)"/ or "rumination (eating)"/ or binge eating/ or food addiction/ 36303

2 (feeding and eating disorders).mp. [mp=title, abstract, heading word, table of contents, key concepts, original title, tests & measures, mesh word] 9219

3 diabulimia.mp. 12

4 (feeding and eating disorders of childhood).mp. [mp=title, abstract, heading word, table of contents, key concepts, original title, tests & measures, mesh word] 229

5 night eating syndrome.mp. 267

6 relative energy deficiency in sport.mp. 6

7 eating disorder*.mp. 35849

8 anorexi*.mp. 19945

9 bulimi*.mp. 14423

10 ARFID.mp. 243

11 avoidant restrictive food intake disorder.mp. 334

12 binge eating.mp. 7879

13 pica.mp. 736

14 rumination disorder.mp. 80

15 rumination syndrome.mp. 27

16 1 or 2 or 3 or 4 or 5 or 6 or 7 or 8 or 9 or 10 or 11 or 12 or 13 or 14 or 15 50512

17 "racial and ethnic groups"/ or african cultural groups/ or asians/ or blacks/ or caribbean cultural groups/ or european cultural groups/ or indigenous populations/ or "latinos/latinas"/ or "middle eastern and north african cultural groups"/ or multiracial/ or "people of color"/ or romanies/ or whites/ or cross cultural differences/ or cultural sensitivity/ or ethnic diversity/ or ethnic values/ or multiculturalism/ or "race (anthropological)"/ or "racial and ethnic attitudes"/ or "racial and ethnic differences"/ or racial disparities/ or racial identity/ or systemic racism/ 206179

18 asians/ or chinese cultural groups/ or japanese cultural groups/ or korean cultural groups/ or south asian cultural groups/ or southeast asian cultural groups/ or vietnamese cultural groups/ 28904

19 japanese cultural groups/ or japanese americans/ 1847

20 southeast asian cultural groups/ or vietnamese cultural groups/ 2159

For peer review only

21 indigenous populations/ or alaska natives/ or american indians/ or inuit/ or pacific islanders/ 15920

22 pacific islanders/ or hawaii natives/ 1152

23 "latinos/latinas"/ or mexican americans/ 33166

24 "middle eastern and north african cultural groups"/ or arabs/ 3975

25 whites/ or anglos/ 21972

26 immigration/ or undocumented immigration/ or expatriates/ or human migration/ or migrant workers/ or refugees/ 41412

27 ethnic minorit*.mp. [mp=title, abstract, heading word, table of contents, key concepts, original title, tests & measures, mesh word] 14105

28 racial minorit*.mp. [mp=title, abstract, heading word, table of contents, key concepts, original title, tests & measures, mesh word] 2386

29 multi-ethnic*.mp. [mp=title, abstract, heading word, table of contents, key concepts, original title, tests & measures, mesh word] 1570

30 BAME.mp. [mp=title, abstract, heading word, table of contents, key concepts, original title, tests & measures, mesh word] 121

31 BIPOC.mp. [mp=title, abstract, heading word, table of contents, key concepts, original title, tests & measures, mesh word] 222

32 people of colo*r.mp. [mp=title, abstract, heading word, table of contents, key concepts, original title, tests & measures, mesh word] 2859

33 17 or 18 or 19 or 20 or 21 or 22 or 23 or 24 or 25 or 26 or 27 or 28 or 29 or 30 or 31 or 32 269191

34 16 and 33 1587

For peer review only

**CINAHL**

Accessibility Information and Tips

Print Search History

EBSCOhost

| # | Query | Limiters/Expanders | Last Run Via | Results |
|---|---|---|---|---|
| S6 | S1 AND S5 | Expanders - Apply equivalent subjects<br>Search modes - Find all my search terms | Interface - EBSCOhost Research Databases<br>Search Screen - Advanced Search<br>Database - CINAHL | 1,018 |
| S5 | S2 OR S3 OR S4 | Expanders - Apply equivalent subjects<br>Search modes - Find all my search terms | Interface - EBSCOhost Research Databases<br>Search Screen - Advanced Search<br>Database - CINAHL | 186,914 |
| S4 | ethnic minority or racial minority or BAME or BIPOC | Expanders - Apply equivalent subjects<br>Search modes - Boolean/Phrase | Interface - EBSCOhost Research Databases<br>Search Screen - Advanced Search<br>Database - CINAHL | 10,473 |
| S3 | (MH "Immigrants") OR (MH "Undocumented Immigrants") OR (MH "Transients and Migrants") | Expanders - Apply equivalent subjects<br>Search modes - Boolean/Phrase | Interface - EBSCOhost Research Databases<br>Search Screen - Advanced Search<br>Database - CINAHL | 23,520 |
| S2 | (MH "Ethnic Groups+") OR (MH "Amish") OR (MH "Arabs") OR (MH "Asians") OR (MH "Cambodians") OR (MH "Chinese") OR (MH "Filipinos") OR (MH "Hmong") OR (MH "Japanese") OR (MH "Koreans") OR (MH "Laotians") OR (MH "Thais") OR (MH "Vietnamese") OR (MH "Black Persons") OR (MH "African Americans") OR (MH "Roma") OR (MH "Hispanic Americans") OR (MH "Mexican Americans") OR (MH "Indigenous Peoples") OR (MH "Aboriginal Canadians") OR (MH "First Nations of Canada") OR (MH "Arctic Peoples") OR (MH "Inuit") OR (MH "First Nations of Australia") OR (MH "Aboriginal Australians") OR (MH "Torres Strait Islanders") OR (MH "Maori") OR (MH "Native Americans") OR (MH "Alaska Natives") OR (MH "Jews") OR | Expanders - Apply equivalent subjects<br>Search modes - Boolean/Phrase | Interface - EBSCOhost Research Databases<br>Search Screen - Advanced Search<br>Database - CINAHL | 166,196 |

For peer review only

| | | | | |
|---|---|---|---|---|
| | (MH "Kurds") OR (MH "White Persons") | | | |
| S1 | (MH "Eating Disorders+") OR (MH "Eating Disorders Management (Iowa NIC)") OR (MH "Binge Eating Disorder") OR (MH "Feeding and Eating Disorders of Childhood+") OR (MH "Bulimia Nervosa") OR (MH "Avoidant Restrictive Food Intake Disorder") OR (MH "Bulimia") OR "eating disorders or anorexia or bulimia or disordered eating or binge eating disorder | Expanders - Apply equivalent subjects<br>Search modes - Boolean/Phrase | Interface - EBSCOhost Research Databases<br>Search Screen - Advanced Search<br>Database - CINAHL | 29,448 |

For peer review only

**Web of Science**

**(TS=("eating disorder" OR anorexia OR bulimia OR "binge eating" OR pica OR "rumination disorder" OR "disordered eating" OR "eating disorders" OR ARFID OR "avoidant restrictive food intake disorder")) AND TS=(ethnicity OR "ethnic group" OR ethnic OR african OR asian OR black OR caribbean OR "central american" OR european OR "middle eastern" OR "north american" OR latino OR oceanian OR australian OR white OR caucasian OR immigrant OR migrant OR refugee OR "racial group" OR "racial minority" OR "ethnic minority" OR multi-ethnic OR BAME OR BIPOC )**

**4993 results**

For peer review only

## Preferred Reporting Items for Systematic reviews and Meta-Analyses extension for Scoping Reviews (PRISMA-ScR) Checklist

| SECTION | ITEM | PRISMA-ScR CHECKLIST ITEM | REPORTED ON PAGE # |
|---|---|---|---|
| **TITLE** | | | |
| Title | 1 | Identify the report as a scoping review. | |
| **ABSTRACT** | | | |
| Structured summary | 2 | Provide a structured summary that includes (as applicable): background, objectives, eligibility criteria, sources of evidence, charting methods, results, and conclusions that relate to the review questions and objectives. | |
| **INTRODUCTION** | | | |
| Rationale | 3 | Describe the rationale for the review in the context of what is already known. Explain why the review questions/objectives lend themselves to a scoping review approach. | |
| Objectives | 4 | Provide an explicit statement of the questions and objectives being addressed with reference to their key elements (e.g., population or participants, concepts, and context) or other relevant key elements used to conceptualize the review questions and/or objectives. | |
| **METHODS** | | | |
| Protocol and registration | 5 | Indicate whether a review protocol exists; state if and where it can be accessed (e.g., a Web address); and if available, provide registration information, including the registration number. | |
| Eligibility criteria | 6 | Specify characteristics of the sources of evidence used as eligibility criteria (e.g., years considered, language, and publication status), and provide a rationale. | |
| Information sources* | 7 | Describe all information sources in the search (e.g., databases with dates of coverage and contact with authors to identify additional sources), as well as the date the most recent search was executed. | |
| Search | 8 | Present the full electronic search strategy for at least 1 database, including any limits used, such that it could be repeated. | |
| Selection of sources of evidence† | 9 | State the process for selecting sources of evidence (i.e., screening and eligibility) included in the scoping review. | |
| Data charting process‡ | 10 | Describe the methods of charting data from the included sources of evidence (e.g., calibrated forms or forms that have been tested by the team before their use, and whether data charting was done independently or in duplicate) and any processes for obtaining and confirming data from investigators. | |
| Data items | 11 | List and define all variables for which data were sought and any assumptions and simplifications made. | |
| Critical appraisal of individual sources of evidence§ | 12 | If done, provide a rationale for conducting a critical appraisal of included sources of evidence; describe the methods used and how this information was used in any data synthesis (if appropriate). | |
| Synthesis of results | 13 | Describe the methods of handling and summarizing the data that were charted. | |

For peer review only

| SECTION | ITEM | PRISMA-ScR CHECKLIST ITEM | REPORTED ON PAGE # |
|---|---|---|---|
| **RESULTS** | | | |
| Selection of sources of evidence | 14 | Give numbers of sources of evidence screened, assessed for eligibility, and included in the review, with reasons for exclusions at each stage, ideally using a flow diagram. | |
| Characteristics of sources of evidence | 15 | For each source of evidence, present characteristics for which data were charted and provide the citations. | |
| Critical appraisal within sources of evidence | 16 | If done, present data on critical appraisal of included sources of evidence (see item 12). | |
| Results of individual sources of evidence | 17 | For each included source of evidence, present the relevant data that were charted that relate to the review questions and objectives. | |
| Synthesis of results | 18 | Summarize and/or present the charting results as they relate to the review questions and objectives. | |
| **DISCUSSION** | | | |
| Summary of evidence | 19 | Summarize the main results (including an overview of concepts, themes, and types of evidence available), link to the review questions and objectives, and consider the relevance to key groups. | |
| Limitations | 20 | Discuss the limitations of the scoping review process. | |
| Conclusions | 21 | Provide a general interpretation of the results with respect to the review questions and objectives, as well as potential implications and/or next steps. | |
| **FUNDING** | | | |
| Funding | 22 | Describe sources of funding for the included sources of evidence, as well as sources of funding for the scoping review. Describe the role of the funders of the scoping review. | |

JBI = Joanna Briggs Institute; PRISMA-ScR = Preferred Reporting Items for Systematic reviews and Meta-Analyses extension for Scoping Reviews.

* Where *sources of evidence* (see second footnote) are compiled from, such as bibliographic databases, social media platforms, and Web sites.

† A more inclusive/heterogeneous term used to account for the different types of evidence or data sources (e.g., quantitative and/or qualitative research, expert opinion, and policy documents) that may be eligible in a scoping review as opposed to only studies. This is not to be confused with *information sources* (see first footnote).

‡ The frameworks by Arksey and O'Malley (6) and Levac and colleagues (7) and the JBI guidance (4, 5) refer to the process of data extraction in a scoping review as data charting.

§ The process of systematically examining research evidence to assess its validity, results, and relevance before using it to inform a decision. This term is used for items 12 and 19 instead of "risk of bias" (which is more applicable to systematic reviews of interventions) to include and acknowledge the various sources of evidence that may be used in a scoping review (e.g., quantitative and/or qualitative research, expert opinion, and policy document).

*From:* Tricco AC, Lillie E, Zarin W, O'Brien KK, Colquhoun H, Levac D, et al. PRISMA Extension for Scoping Reviews (PRISMAScR): Checklist and Explanation. Ann Intern Med. 2018;169:467–473. doi: 10.7326/M18-0850.



For peer review only - http://bmjopen.bmj.com/site/about/guidelines.xhtml

BMJ Open

# A protocol for a scoping review to identify research reporting on eating disorders in minority ethnic populations in the UK, Canada, Australia, and New Zealand


| | |
|---|---|
| Journal: | *BMJ Open* |
| Manuscript ID | bmjopen-2023-075034.R2 |
| Article Type: | Protocol |
| Date Submitted by the Author: | 19-Jan-2024 |



| | |
|---|---|
| Complete List of Authors: | Tuomainen, Helena; University of Warwick<br>McGowan, Rose; University of Warwick<br>Williams-Ridgway, Aliyah; University of Warwick<br>Guy, Katie; Birmingham Women's and Children's Hospitals NHS Foundation Trust<br>McNeil, Sheryllin; Birmingham Women's and Children's Hospitals NHS Foundation Trust |



| | |
|---|---|
| <b>Primary Subject Heading</b>: | Mental health |
| Secondary Subject Heading: | Health services research |
| Keywords: | Eating disorders < PSYCHIATRY, Health Services Accessibility, Systematic Review |
| | |


SCHOLARONE™
Manuscripts

# A protocol for a scoping review to identify research reporting on eating disorders in minority ethnic populations in the UK, Canada, Australia, and New Zealand

Tuomainen H[1], McGowan R[1], Williams-Ridgway A[1], Guy K[2], McNeil S[2]

[1]Warwick Medical School, University of Warwick, Coventry, UK

[2]Birmingham Women's and Children's NHS Foundation Trust, Birmingham, UK

helena.tuomainen@warwick.ac.uk

Rose.McGowan@warwick.ac.uk

aliyah.williams-ridgway@warwick.ac.uk

katie.guy@nhs.net

sheryllin.mcneil1@nhs.net

Corresponding author:

Helena Tuomainen
Warwick Medical School
University of Warwick
CV47AL
helena.tuomainen@warwick.ac.uk
+44-07722-330979

Keywords: Eating disorders, Population groups, Ethnicity, treatment, healthcare disparities



For peer review only - http://bmjopen.bmj.com/site/about/guidelines.xhtml

# Abstract

### *Introduction*

Eating disorders (EDs) are common, severe and often life threatening psychiatric conditions. Notwithstanding stereotypes, EDs affect individuals from all racial and ethnic backgrounds. However, despite similar and in some cases increased prevalence of disordered eating and EDs among minority ethnic groups there appear to be disparities when it comes to ED diagnosis and treatment. To date, most of the existing literature exploring disordered eating and EDs among minority ethnic groups has been conducted in the United States (US). The present scoping review aims to examine the extent, range and nature of research activity into disordered eating and EDs in the UK, Canada, Australia and New Zealand providing a comprehensive overview of the existing literature. A special focus will be placed on studies exploring prevalence, access to care and treatment experiences.

### *Methods and Analysis*

The scoping review framework first outlined by Arksey and O'Malley (2005) and improved upon by Levac et al. (2010) was used to guide the development of this scoping review protocol. A detailed systematic search of relevant databases (Medline, EMBASE, PsycInfo, CINAHL, and Web of Science) will be conducted. Relevant literature will also be identified from the reference lists of included studies. Two reviewers will independently screen titles and abstracts and full-text studies for inclusion using specified inclusion and exclusion criteria. A third reviewer will resolve disagreements if necessary. Relevant data will be extracted using a data charting form. Quantitative and qualitative summaries of extracted data will be provided.

### *Ethics and Dissemination*

No ethical approval is required for this study. Findings will be of benefit to researchers, clinicians and policy makers by highlighting areas for future research and identifying ways to making ED treatment more accessible for individuals from all backgrounds. Findings will be disseminated via conferences, presentations and peer-reviewed journals.


# Strengths and limitations of this study

- This protocol will ensure transparency in methodology to reduce the likelihood of reviewing bias.
- Studies conducted in non-Western, low- or middle-income countries are not included in this review.



For peer review only - http://bmjopen.bmj.com/site/about/guidelines.xhtml
For peer review only

- There will be no quality assessment of the included studies.
- Only studies published in English will be included.

# Introduction

## Background

Eating disorders (EDs) are complex psychiatric conditions characterised by disordered eating and/or weight-control behaviours [1] which typically have their onset during adolescence [2]. A recent systematic review reported a pooled global lifetime and 12-month prevalence of EDs of 0.91% and 0.43%, respectively [3]. EDs have profound physical and psychological impacts on the affected individual and those around them. EDs have high co-morbidity with other psychiatric conditions and are associated with suicidal thoughts and attempts [4,5]. The medical complications that often accompany EDs involve almost all organ systems and can be life-threatening [6], leading to significantly increased mortality rates [7]. Given the negative consequences of EDs, it is imperative that we understand their prevalence and treatment within and across ethnic groups.

Despite the prevailing stereotype that EDs only affect "skinny, White, affluent girls" (the SWAG stereotype) [8], it is widely reported that EDs affect individuals from all racial and ethnic backgrounds [9]. However, findings regarding the prevalence of disordered eating and EDs across ethnic groups are mixed. Some studies report no difference in disordered eating and ED prevalence across different ethnic groups [10-12]. Others, however, have reported higher prevalence of disordered eating among minority ethnic men and women relative to White individuals [13]. In contrast, some studies have found higher prevalence of EDs in White adults when compared to other racial and ethnic groups [8,14,15]. Reported prevalence seems to vary based on the racial or ethnic group and type of disordered eating or ED being studied [9]. Differences in study design and diagnostic criteria used may also contribute to differences in reported prevalence [11].

Despite similar and in some cases increased rates of disordered eating and EDs among minority ethnic groups there appear to be disparities when it comes to ED diagnosis and treatment. In their systematic review, Sinha and Warfa [16] found minority ethnic individuals in the UK and US were less likely to seek and receive ED treatment as well as less frequently diagnosed and referred to specialist ED services. Since publication of this review further studies have replicated these findings [8,17,18]. Such disparities in diagnosis and treatment have been attributed to a lack of perceived need for treatment among these populations [8], differences in symptom presentation [18] and culturally insensitive diagnostic criteria [19].



For peer review only

For peer review only - http://bmjopen.bmj.com/site/about/guidelines.xhtml

To date, most of the existing literature exploring disordered eating and EDs among minority ethnic groups has been conducted in the United States (US). This is apparent in recent narrative and systematic reviews which have explored the identification, prevalence, presentation, risk factors and treatment of disordered eating and EDs among minority racial and ethnic populations [9,16,20-23]. The overwhelming majority of studies included in these reviews have been conducted in the US.

Researchers have called for more research exploring ethnic and racial diversity in EDs to be conducted outside of the US [24]. Generalisability of findings from US studies to countries with different healthcare systems and sociocultural and environmental contexts has been questioned [11,25]. Franko and Rodgers [26] also argue research outside of the US is necessary to fully understand the extent of similarities and differences within and across ethnic groups.

## Rationale

The present scoping review aims to identify and describe studies reporting on disordered eating and EDs in minority ethnic groups in the UK, Canada, Australia and New Zealand; to our knowledge this will be the first review to do so. These countries have large established minority ethnic populations due to high levels of immigration and/or the forced displacement of Indigenous peoples, as well as broadly comparable healthcare systems. By providing a broad overview of the extent, nature and range of existing research conducted in these countries we hope to elucidate any knowledge gaps to inform future research. This is crucial considering the identified need for more research exploring racial and ethnic differences in EDs outside of the US [25].

Given the well-documented racial and ethnic health disparities in the diagnosis and treatment of EDs [16] and mental health conditions more broadly [27], a special focus will be placed on studies which have reported on the prevalence of disordered eating and EDs, access to care and treatment experiences among minority ethnic groups. Such studies will be particularly relevant in identifying any health disparities in the countries of focus and highlighting possible ways to overcome these. This is of particular importance given prompt diagnosis and intervention can improve ED treatment outcomes and recovery prospects [28].

## Objectives

In this scoping review we aim to examine the extent, range and nature of research activity into disordered eating and EDs among minority ethnic populations in the UK, Canada, Australia and New Zealand. The specific objectives are to:



For peer review only - http://bmjopen.bmj.com/site/about/guidelines.xhtml

1. Map the literature according to the main themes/topics addressed, target populations and methodologies utilised;
2. Report on findings regarding ED prevalence, access to care and treatment experiences;
3. Identify gaps and/or limitations in the literature and suggest areas for future research.

# Methods and Analysis

## Protocol Design

A scoping review was chosen as the most appropriate methodology. Scoping reviews are suited to answering broad research questions and are designed to identify the types of evidence available in a field, examine how this research has been conducted and highlight any knowledge gaps [29]. The present scoping review will follow the five stage framework first outlined by Arksey and O'Malley [30] and subsequently improved by Levac et al [31]. The stages are as follows: (1) identifying the research questions; (2) identifying the relevant literature; (3) selecting studies; (4) charting the data; and (5) collating, summarising and reporting the results. To ensure quality of the scoping review, the development and reporting of this protocol followed the Preferred Reporting Items for Systematic reviews and Meta-Analyses Extension for Scoping Reviews (PRISMA-ScR) guidelines [32]. The protocol for this scoping review is also registered on OSF (https://osf.io/w3d6g). Any changes made as part of the iterative nature of the scoping review will be documented here.

## Stage 1: Identifying the Research Questions

The first stage of a scoping review is to develop research questions informed by the purpose and objectives of the scoping review[31]. Based on the aforementioned objectives of this scoping review we have formulated six research questions. These are described in further detail below. Given the iterative nature of scoping reviews, a reflexive approach will be adopted. If necessary, as we become more familiar with the existing literature our research questions will be refined and modified accordingly.

**Question 1: What topics are being addressed by the current literature?**

Within the US studies have explored the identification, prevalence, presentation, risk factors and treatment of EDs among minority racial and ethnic groups. For each study included in this scoping review we will report on the primary focus of the study. By doing so we hope to provide a summary of the themes/topics that have been addressed by the current literature on disordered eating and EDs among minority ethnic populations in the UK, Canada, Australia and New Zealand.



For peer review only - http://bmjopen.bmj.com/site/about/guidelines.xhtml

**Question 2: How has this research been conducted?**

This research question aims to explore how the included studies have been designed and conducted. For each study we will report the research design and methodology used to answer research questions.

**Question 3: Which populations have been targeted for this research?**

By answering this question we hope to provide an overview of which groups and populations have been studied. We will report on key participant characteristics (e.g., age, gender, socioeconomic status, co-morbidities) and examine which ethnic groups and types of disordered eating or EDs have been studied. We also aim to explore how and where participants were recruited (e.g., sampling technique, clinical or non-clinical sample, sample size).

**Question 4: Are there differences in reported ED prevalence across ethnicity?**

This question aims to assess whether reported prevalence rates of disordered eating and EDs differ across ethnic groups.

**Question 5: Are there differences in access to ED treatment across ethnic groups?**

To answer this question we will extract and analyse data from studies which have reported findings regarding any of the following: referrals to ED services, service utilisation, barriers to care or help-seeking (or treatment-seeking).

**Question 6: Do ED treatment experiences differ across ethnic groups?**

The purpose of this question is to assess what is currently known regarding the treatment experiences of minority ethnic patients. We will aim to explore how minority ethnic individuals and/or stakeholders involved in the delivery or receipt of their treatment (e.g., clinicians, family members) describe their experiences of treatment. We will also aim to identify the types of treatment that have been used in minority ethnic populations.

## Stage 2: Identifying Relevant Studies

**Information Sources**

The second stage of a scoping review is to identify relevant studies for selection and data extraction. This will involve a systematic search of the following electronic databases: (1) Medline (2) PsycINFO; (3) EMBASE; (4) CINAHL and (5) Web of Science. To ensure the search is as comprehensive as possible, backwards citation chaining of included studies will be used to identify any additional studies of relevance. For sake of completeness, a web search using Google Scholar will also be completed. Both published and grey literature (e.g., theses) will be considered for inclusion.



For peer review only - http://bmjopen.bmj.com/site/about/guidelines.xhtml

**Search Strategy**

The search strategy was developed by members of the research team (HT, SM, and RM) in collaboration with a specialty academic support librarian. Search terms include keywords and terms related to (1) EDs and (2) ethnicity. See online supplementary appendix 1 for the search strategy for each of the databases. No date limits will be applied to the search results, but the database search query will be set to produce articles in English only due to resource constraints.

The search results for each database will be documented and references will be imported into the citation management system Endnote, where duplicates will be removed before studies are screened for inclusion.

## Stage 3: Selecting Studies

The process of selecting studies will consist of two stages: (1) title and abstract screening and (2) full-text screening. Each stage will involve two data reviewers independently screening the identified studies against the inclusion and exclusion criteria. All screening will be conducted using the online screening tool Rayyan.

In the first round of screening two reviewers (RM and HT) will review paper titles and abstracts. Papers will be catagorised as either 'include', 'exclude' or 'maybe'. Decisions as to whether papers are eligible for full-text review will be made as follows: (1) if both reviewers agree to 'include', the study will move on to the second screening; (2) if both reviewers agree to 'exclude', the study will not be read in full and will be excluded from the review; (3) if the reviewers are not unanimous in their agreement, the study will also move onto the second screening to be read in full before a final decision is made. Where a paper abstract is unavailable, papers will be assigned to the 'maybe' category and automatically move on to the second stage of screening to be read in full. Eligibility criteria will be further refined and finalised following the first round of screening if necessary.

In the second round of screening two reviewers (AWR and HT) will read the remaining articles in full to determine whether they meet the inclusion and exclusion criteria. If the decision to include or exclude if not unanimous, both reviewers will assess the study together and discuss disagreements. A third reviewer may be consulted for arbitration of any conflicting decisions. A flowchart outlining decisions made regarding study inclusion will be produced using the PRIMSA template [33].

**Eligibility Criteria**

Eligibility criteria were developed using the population, context, concept (PCC) framework [34] (see table 1). Inclusion and exclusion criteria are further detailed below (see table 2):



For peer review only - http://bmjopen.bmj.com/site/about/guidelines.xhtml

**Table 1**

Core elements of the scoping review based on Population Concept, Context (PCC) Framework

| | | |
|---|---|---|
| **Population** | Minority Ethnic Groups | A group of people within a country that: "is numerically smaller than the rest of the population; is not in a dominant position; has a culture, language, religion or race that is distinct from that of the majority; and its members have a will to preserve those characteristics" [35 as cited in 36, p.97]. |
| **Concept** | Eating Disorders | Clinical psychiatric disorders, including anorexia nervosa, bulimia nervosa and binge eating disorder, characterised by disturbed eating behaviours, body image concerns and unhealthy weight control behaviours[1]. |
| | Disordered Eating | Subclinical levels of unhealthy eating behaviours including restrictive eating, binge eating, unhealthy weight control behaviours [37]. |
| **Context** | Studies conducted in the UK, Canada, Australia or New Zealand. | |

For peer review only

**Table 2**

Inclusion and Exclusion Criteria

| | Inclusion | Exclusion |
|---|---|---|
| Population | - Sub- or full sample of minority ethnic participants or data related to minority ethnic individuals<br>- Where multiple ethnic groups are included in a sample the findings are reported according to ethnicity. For example, ethnicity is the main predictor variable in analyses or the paper reports on ethnic differences in study outcomes (e.g., prevalence rates, scores on psychometric measures, referral rates etc.).<br>- All ages | - Where multiple minority ethnic groups are included in the sample, but authors did not report any outcomes or analysis according to ethnicity (e.g., ethnicity only reported in sample demographics, analyses reported for sample as a whole rather than individual ethnic groups, where analyses are controlled or adjusted for ethnicity but no further findings or data relating to ethnicity were reported). |
| Concept | - Study focuses on clinical EDs (i.e., meeting DSM or ICD criteria as assessed using clinician interview or a validated psychometric measure) or disordered eating (i.e., assessed with a validated psychometric measure). | - Focuses solely on risk factors, associated concepts or related conditions (e.g., food addiction, obesity, body image).<br>- Focuses solely on ARFID, pica or rumination disorder. |
| Context | - Study conducted in UK, Canada, Australia or New Zealand. | |
| Study Design | - Any study design (e.g, case reports, case studies, cohort studies, randomized controlled trials etc.) | |



For peer review only - http://bmjopen.bmj.com/site/about/guidelines.xhtml

| | | |
|---|---|---|
| Publication Type | - Peer reviewed journal articles or theses | - Review articles, editorials, commentaries, letters, newsletters, opinion or reflection pieces, book chapters, book reviews, poster presentations or conference abstracts. |
| Language | - Published in English | |

## Stage 4: Charting the Data/Data Extraction

Once studies have been identified for final inclusion, data from these studies will be extracted and charted. A comprehensive data charting form will be developed in Excel and used to extract relevant information from studies. Two reviewers (AWR and HT) will pilot the data charting form on a sample of randomly selected papers (10% of included papers). Any discrepancies or uncertainties highlighted by this exercise will be discussed and the charting form will be adjusted accordingly. One reviewer (AWR) will extract the data from the remaining papers. The research team will meet regularly to discuss progress throughout the data extraction process and further changes to the data extraction form will be made as needed in an iterative process. For example, additional categories of interest may emerge. Any changes made and the rationale for doing so will be reported in final scoping review findings.

Data to be extracted will include some standard information about the paper. For example, study title, author(s), year of publication and country of publication. Additional information specific to our research questions will also be extracted. This will include the study aims/objectives, research focus/topic, study design and methodology, sample characteristics and demographics (e.g., age, gender, ED diagnosis, ethnicity, sample size), setting in which the study was conducted (e.g., clinical or non-clinical setting), summary of relevant findings, and recommendations of the author(s) if applicable. In line with guidance on conducting scoping review, there will be no critical appraisal of the included studies [32].

## Stage 5: Collating, Summarising and Reporting the Results

Rather than meta-synthesis or critical appraisal of studies, the purpose of a scoping review is to provide an overview of the existing literature, summarising what is already known and identifying any knowledge gaps [29]. The extracted data will be summarised in a way that maps the extent, range and



For peer review only - http://bmjopen.bmj.com/site/about/guidelines.xhtml

nature of research into disordered eating and EDs among minority ethnic populations in the UK, Canada, Australia and New Zealand, whilst placing particular focus on differences in prevalence, access to care and treatment experiences across ethnicity.

We will provide a descriptive numerical summary of the characteristics of included studies. Studies will be classified and grouped according to their characteristics. Extracted data will be presented in tables, charts and visual maps. To accompany tabular and visual summaries, we will also provide a qualitative narrative summary of the data and how it relates to our research questions. If data and resources permit, qualitative findings relating to treatment experiences of minority ethnic individuals will be synthesised following Thomas and Harden's [38] thematic synthesis approach. Thematic synthesis adapts the key principles of thematic analysis [39] for use in reviews. The aim of the analysis is to identify and interpret themes/patterns across papers. This is done across three stages (1) line by line coding of the results section; (2) organising codes into descriptive themes; and (3) generation of analytical themes [38]. If conducted, further details on this analysis procedure will be provided in the final review. Finally, informed by any significant gaps and/or limitations of the literature we will recommend avenues for future research. The scoping review findings will also be discussed in relation to clinical practice and policy. Reporting of the results will follow PRISMA ScR guidelines [32].

## Ethics and Dissemination

As scoping reviews involve the methodological examination and integration of existing literature and resources ethical approval is not required. Once all stages of the scoping review are complete, findings will be disseminated via conferences, presentations and the submission of the final review for peer-reviewed publication.

## Patient and Public Involvement

Patients or the public have not been involved in the design, conduct, reporting, or dissemination plans of this scoping review.

## Acknowledgements

The authors thank Samantha Johnson, Academic Support Librarian, for assisting in the search strategy.



For peer review only - http://bmjopen.bmj.com/site/about/guidelines.xhtml

## Author Contributions

HT and SM were involved in the initial conception of the review. HT is responsible for the design of the review. RM aided with the design of the review and drafted the first manuscript. AWR revised the manuscript making critical edits. KG contributed meaningfully to the drafting and editing of the manuscript. All authors read and approved the final manuscript.

## Funding Statement

This research is funded by the National Institute for Health and Care Research (NIHR) Applied Research Collaboration (ARC) West Midlands (Grant number NIHR200165). The views expressed are those of the authors and not necessarily those of the NIHR or the Department of Health and Social Care.

## Competing Interests Statement

None declared.

## Word Count

2545

For peer review only



For peer review only - http://bmjopen.bmj.com/site/about/guidelines.xhtml

# References

1. American Psychiatric Association. Diagnostic and Statistical Manual of Mental Sisorders (5th edn.) APA, 2013.

2. Volpe U, Tortorella A, Manchia M, et al. Eating disorders: What age at onset? Psychiatry Res. 2016;238:225-7.

3. Qian J, Wu Y, Liu F, et al. An update on the prevalence of eating disorders in the general population: a systematic review and meta-analysis. Eating and Weight Disorders-Studies on Anorexia, Bulimia and Obesity. 2021; 27(2):415-428.

4. Chamay-Weber C, Narring F, Michaud PA. Partial eating disorders among adolescents: A review. J of Adolesc Health. 2005;37(5):416-26.

5. Lewinsohn PM, Striegel-Moore RH, Seeley JR. Epidemiology and natural course of eating disorders in young women from adolescence to young adulthood. J Am Academy of Child Adolesc Psychiatry. 2000;39(10):1284-92.

6. Voderholzer U, Haas V, Correll CU, et al. Medical management of eating disorders: an update. Curr Opin Psychiatry. 2020;33(6):542-53.

7. van Hoeken D, Hoek HW. Review of the burden of eating disorders: mortality, disability, costs, quality of life, and family burden. Curr Opin Psychiatry. 2020;33(6):521-27.

8. Sonneville K, Lipson S. Disparities in eating disorder diagnosis and treatment according to weight status, race/ethnicity, socioeconomic background, and sex among college students. Int J Eat Disord. 2018;51(6):518-26.

9. Rodgers RF, Berry R, Franko DL. Eating disorders in ethnic minorities: An update. Curr Psychiatry Rep. 2018;20:1-11.

10. Cheng ZH, Perko VL, Fuller-Marashi L, et al. Ethnic differences in eating disorder prevalence, risk factors, and predictive effects of risk factors among young women. Eat Behav. 2019;32:23-30.



For peer review only - http://bmjopen.bmj.com/site/about/guidelines.xhtml

11. Solmi F, Hotopf M, Hatch S, et al. Eating disorders in a multi-ethnic inner-city UK sample: prevalence, comorbidity and service use. Soc Psychiatry Psychiatr Epidemiol. 2016;51(3):369-81.

12. Marques L, Alegria M, Becker AE, et al. Comparative prevalence, correlates of impairment, and service utilization for eating disorders across US ethnic groups: Implications for reducing ethnic disparities in health care access for eating disorders. Int J Eat Disord. 2011;44(5):412-20.

13. Simone M, Telke S, Anderson LM, et al. Ethnic/racial and gender differences in disordered eating behavior prevalence trajectories among women and men from adolescence into adulthood. Soc Sci Med. 2022;294:114720.

14. Udo T, Grilo CM. Prevalence and correlates of DSM-5–defined eating disorders in a nationally representative sample of US adults. Biol Psychiatry. 2018;84(5):345-54.

15. Striegel-Moore RH, Dohm FA, Kraemer HC, et al. Eating disorders in white and black women. Am J Psychiatry. 2003;160(7):1326-31.

16. Sinha S, Warfa N. Treatment of eating disorders among ethnic minorities in western settings: a systematic review. Psychiatr Danub. 2013;25(2):295-9.

17. Coffino JA, Udo T, Grilo CM. Rates of help-seeking in US adults with lifetime DSM-5 eating disorders: prevalence across diagnoses and differences by sex and ethnicity/race. Mayo Clin Proc. 2019;94(8):1415-1426.

18. Solmi F, Hatch SL, Hotopf M, et al. Prevalence and correlates of disordered eating in a general population sample: the South East London Community Health (SELCoH) study. Soc Psychiatry Psychiatr Epidemiol. 2014;49(8):1335-46.

19. Lee-Winn A, Mendelson T, Mojtabai R. Racial/ethnic disparities in binge eating: disorder prevalence, symptom presentation, and help-seeking among Asian Americans and non-Latino Whites. Am J Public Health. 2014;104(7):1263-5.

20. Goode RW, Cowell MM, Mazzeo SE, et al. Binge eating and binge-eating disorder in Black women: A systematic review. Int J Eat Disord. 2020;53(4):491-507.

For peer review only



For peer review only - http://bmjopen.bmj.com/site/about/guidelines.xhtml

21. Acle A, Cook BJ, Siegfried N, et al. Cultural considerations in the treatment of eating disorders among racial/ethnic minorities: A systematic review. J Cross Cult Psychol. 2021;52(5):468-88.

22. Song S, Stern CM, Deitsch T, et al. Acculturation and eating disorders: a systematic review. Eating and Weight Disorders-Studies on Anorexia, Bulimia and Obesity. 2023;28(1):39.

23. Doris E, Shekriladze I, Javakhishvili N, et al. Is cultural change associated with eating disorders? A systematic review of the literature. Eating and Weight Disorders-Studies on Anorexia, Bulimia and Obesity. 2015;20(2):149-60.

24. Egbert AH, Hunt RA, et al. Reporting racial and ethnic diversity in eating disorder research over the past 20 years. Int J Eat Disord. 2022;55(4):455-62.

25. Mikhail ME, Klump KL. A virtual issue highlighting eating disorders in people of black/African and Indigenous heritage. Int J Eat Disord. 2021;54(3):459-67.

26. Franko DL, Rodgers RF. Ethnicity and eating disorders: More similarities than differences. In: Wade T, Wildes J, editors. Encyclopedia of Feeding and Eating Disorders. New York, NY: Springer; 2017. p. 366-70.

27. Maguire TG, Miranda J. Racial and ethnic disparities in mental healthcare: evidence and policy implications. Health Aff. 2008;27(2):393-403.

28. Linardon J, Messer M, Rodgers RF, et al. A systematic scoping review of research on COVID-19 impacts on eating disorders: A critical appraisal of the evidence and recommendations for the field. Int J Eat Disord. 2022;55(1):3-8.

29. Munn Z, Peters MD, Stern C, et al. Systematic review or scoping review? Guidance for authors when choosing between a systematic or scoping review approach. BMC Med Res Methodol. 2018;18:1-7.

30. Arksey H, O'Malley L. Scoping studies: towards a methodological framework. Int J Soc Res Methodol. 2005;8(1):19-32.



For peer review only - http://bmjopen.bmj.com/site/about/guidelines.xhtml

31. Levac D, Colquhoun H, O'Brien KK. Scoping studies: advancing the methodology. Implementation Sci. 2010;5:1-9.

32. Tricco AC, Lillie E, Zarin W, et al. PRISMA extension for scoping reviews (PRISMA-ScR): checklist and explanation. Ann Internal Med. 2018;169(7):467-73.

33. Moher D, Liberati A, Tetzlaff J, et al. Preferred reporting items for systematic reviews and meta-analyses: the PRISMA statement. Ann Internal Med. 2009;151(4):264-9.

34. Aromataris E, Munn Z, eds. JBI manual for evidence synthesis [e-book]. JBI; 2020. https://synthesismanual.jbi.global [accessed 11 Dec 2023].

35. Foa, RS. Creating an Inclusive Society: Evidence from Social Indicators and Trends. [online]. 2015. https://www.repository.cam.ac.uk/handle/1810/342924 [accessed 11 Dec 2023].

36. United Nations. Indigenous Peoples and Ethnic Minorities: Marginalization Is the Norm. In: The Report on the World Social Situation. New York, NY: United Nations Publication; 2018. p. 97-108.

37. Krentz EM, Warschburger P. Sports-related correlates of disordered eating in aesthetic sports. Psychol of Sport Exerc. 2011;12(4):375-82.

38. Thomas J, Harden A. Methods for the thematic synthesis of qualitative research in systematic reviews. BMC Med Res Methodol. 2008;45:8(1):1-10.

39. Braun V, Clarke V. Using thematic analysis in psychology. Qual Res Psychol. 2006;3(2):77-101.



For peer review only - http://bmjopen.bmj.com/site/about/guidelines.xhtml

Database Searches

**Medline**

Ovid MEDLINE(R) ALL <1946 to March 30, 2023>

1 "feeding and eating disorders"/ or anorexia nervosa/ or avoidant restrictive food intake disorder/ or binge-eating disorder/ or bulimia nervosa/ or diabulimia/ or "feeding and eating disorders of childhood"/ or food addiction/ or night eating syndrome/ or orthorexia nervosa/ or pica/ or relative energy deficiency in sport/ or female athlete triad syndrome/ or rumination syndrome/ 35804

2 eating disorder*.mp. 33463

3 anorexi*.mp. 42181

4 bulimi*.mp. 12324

5 ARFID.mp. 268

6 avoidant restrictive food intake disorder.mp. 432

7 binge eating.mp. 7407

8 pica.mp. 3495

9 rumination disorder.mp. 41

10 rumination syndrome.mp. 162

11 1 or 2 or 3 or 4 or 5 or 6 or 7 or 8 or 9 or 10 73164

12 population groups/ or african people/ or north african people/ or sub-saharan african people/ or central african people/ or east african people/ or southern african people/ or west african people/ or asian people/ or asian/ or central asian people/ or east asian people/ or north asian people/ or southeast asian people/ or west asian people/ or middle eastern people/ or south asian people/ or black people/ or "black or african american"/ or caribbean people/ or central american people/ or indians, central american/ or european people/ or eastern european people/ or "scandinavians and nordic people"/ or "middle eastern and north africans"/ or north american people/ or "american indian or alaska native"/ or indians, north american/ or alaskan natives/ or indigenous canadians/ or inuit/ or navajo people/ or pima people/ or population groups, us/ or ethnicity/ or "hispanic or latino"/ or mexican americans/ or racial groups/ or "native hawaiian or other pacific islander"/ or white/ or oceanians/ or australasian people/ or "australian aboriginal and torres strait islander peoples"/ or pacific island people/ or maori people/ or south american people/ or indians, south american/ or white people/ 326840

13 "emigrants and immigrants"/ or undocumented immigrants/ or refugees/ or "transients and migrants"/ 39912

14 "black or african american"/ or amish/ or arabs/ or "asian american native hawaiian and pacific islander"/ or asian/ or "native hawaiian or other pacific islander"/ or "hispanic or latino"/ or mexican americans/ or indigenous peoples/ or "american indian or alaska native"/ or indians, central american/ or indians, north american/ or alaskan natives/ or indigenous canadians/ or inuit/ or

For peer review only

navajo people/ or pima people/ or indians, south american/ or "australian aboriginal and torres strait islander peoples"/ or maori people/ or roma/ 133629

15 asia, southern/ or afghanistan/ or bangladesh/ or bhutan/ or india/ or sikkim/ or maldives/ or nepal/ or pakistan/ or sri lanka/ 172233

16 ethnic minorit*.mp. 15411

17 racial minorit*.mp. 2337

18 multi-ethnic*.mp. 5370

19 "ethnic and racial minorities"/ or minority groups/ 17800

20 BAME.mp. 358

21 BIPOC.mp. 264

22 people of colo*r.mp. 1373

23 12 or 13 or 14 or 15 or 16 or 17 or 18 or 19 or 20 or 21 or 22 549059

24 11 and 23 1312

For peer review only

**EMBASE**

Embase Classic+Embase <1947 to 2023 Week 12>

1 eating disorder/ or anorexia nervosa/ or avoidant restrictive food intake disorder/ or binge eating disorder/ or bulimia/ or emotional eating/ or food addiction/ or food aversion/ or food neophobia/ or food refusal/ or muscle dysmorphia/ or orthorexia/ or pica/ or purging disorder/ or relative energy deficiency in sport/ 64869

2 (feeding and eating disorders).mp. [mp=title, abstract, heading word, drug trade name, original title, device manufacturer, drug manufacturer, device trade name, keyword heading word, floating subheading word, candidate term word]5597

3 diabulimia.mp. 64

4 (feeding and eating disorders of childhood).mp. [mp=title, abstract, heading word, drug trade name, original title, device manufacturer, drug manufacturer, device trade name, keyword heading word, floating subheading word, candidate term word] 52

5 night eating syndrome.mp. 465

6 rumination syndrome/ or feeding disorder/ 6564

7 eating disorder*.mp. [mp=title, abstract, heading word, drug trade name, original title, device manufacturer, drug manufacturer, device trade name, keyword heading word, floating subheading word, candidate term word]47823

8 anorexi*.mp. [mp=title, abstract, heading word, drug trade name, original title, device manufacturer, drug manufacturer, device trade name, keyword heading word, floating subheading word, candidate term word] 115739

9 bulimi*.mp. [mp=title, abstract, heading word, drug trade name, original title, device manufacturer, drug manufacturer, device trade name, keyword heading word, floating subheading word, candidate term word] 18145

10 ARFID.mp. [mp=title, abstract, heading word, drug trade name, original title, device manufacturer, drug manufacturer, device trade name, keyword heading word, floating subheading word, candidate term word] 438

11 avoidant restrictive food intake disorder.mp. [mp=title, abstract, heading word, drug trade name, original title, device manufacturer, drug manufacturer, device trade name, keyword heading word, floating subheading word, candidate term word] 698

12 binge eating.mp. [mp=title, abstract, heading word, drug trade name, original title, device manufacturer, drug manufacturer, device trade name, keyword heading word, floating subheading word, candidate term word] 12511

13 pica.mp. [mp=title, abstract, heading word, drug trade name, original title, device manufacturer, drug manufacturer, device trade name, keyword heading word, floating subheading word, candidate term word] 4755

For peer review only

14 rumination disorder.mp. [mp=title, abstract, heading word, drug trade name, original title, device manufacturer, drug manufacturer, device trade name, keyword heading word, floating subheading word, candidate term word]57

15 rumination syndrome.mp. [mp=title, abstract, heading word, drug trade name, original title, device manufacturer, drug manufacturer, device trade name, keyword heading word, floating subheading word, candidate term word]414

16 1 or 2 or 3 or 4 or 5 or 6 or 7 or 8 or 9 or 10 or 11 or 12 or 13 or 14 or 15 165879

17 population group/ or ancestry group/ or citizen group/ or ethnic group/ or minority group/ 107708

18 ancestry group/ or asian american/ or asian continental ancestry group/ or australoid/ or black person/ or british asian/ or caucasian/ or hispanic/ or indigenous people/ or migrant/ or mongoloid/ or multiracial person/ or oceanic ancestry group/ 279452

19 black person/ or african american/ or african brazilian/ or african caribbean/ 144776

20 caucasian/ or european american/ 172363

21 hispanic/ or mexican american/ 91634

22 indigenous people/ or alaska native/ or american indian/ or canadian aboriginal/ or first nation/ or indigenous australian/ or taiwanese aborigine/ 35946

23 migrant/ or emigrant/ or forced migrant/ or immigrant/ or migrant worker/ 34289

24 multiracial person/ 711

25 oceanic ancestry group/ or pacific islander/ or torres strait islander/ 10630

26 citizen group/ or african/ or asian/ or "caribbean (person)"/ or central american/ or european/ or north american/ or oceanian/ or south american/ 255326

27 african/ or central african/ or east african/ or north african/ or southern african/ or west african/ 11807

28 central african/ or angolan/ or cameroonian/ or "chadian (citizen)"/ or "citizen of the central african republic"/ or "congolese (brazzaville)"/ or "congolese (kinshasa)"/ or gabonese/ 855

29 east african/ or eritrean/ or ethiopian/ or kenyan/ or "malagasy (citizen)"/ or malawian/ or mauritian/ or mozambican/ or rwandan/ or "somali (citizen)"/ or tanzanian/ or ugandan/ or zambian/ or zimbabwean/ 6458

30 north african/ or algerian/ or egyptian/ or libyan/ or moroccan/ or sudanese/ or tunisian/ 11694

31 southern african/ or namibian/ or south african/ or "swazi (citizen)"/ 4278

32 west african/ or beninese/ or burkinabe/ or gambian/ or ghanaian/ or guinean/ or "ivorian (citizen)"/ or liberian/ or malian/ or mauritanian/ or nigerian/ or nigerien/ or senegalese/ or sierra leonean/ or togolese/ 5473

33 asian/ or central asian/ or east asian/ or south asian/ or southeast asian/ or west asian/ 106199

For peer review only

34 central asian/ or kazakhstani/ or "kyrgyz (citizen)"/ or "tajik (citizen)"/ or "turkmen (citizen)"/ or "uzbek (citizen)"/ 405

35 east asian/ or chinese/ or "japanese (citizen)"/ or "mongolian (citizen)"/ or north korean/ or south korean/ or taiwanese/ 93841

36 south asian/ or afghan/ or bangladeshi/ or bhutanese/ or indian/ or nepalese/ or pakistani/ or sri lankan/ 51986

37 southeast asian/ or burmese/ or cambodian/ or "filipino (citizen)"/ or indonesian/ or laotian/ or malaysian/ or singaporean/ or "thai (citizen)"/ or vietnamese/11375

38 west asian/ or "armenian (citizen)"/ or azerbaijani/ or bahraini/ or cypriot/ or emirati/ or "georgian (citizen)"/ or "iranian (citizen)"/ or iraqi/ or israeli/ or jordanian/ or kuwaiti/ or lebanese/ or omani/ or palestinian/ or qatari/ or saudi/ or syrian/ or turkish citizen/ or yemeni/ 16521

39 caribbean/ or antillean/ 5271

40 central american/ or belizean/ or costa rican/ or guatemalan/ or honduran/ or mexican/ or nicaraguan/ or panamanian/ or salvadoran/ 6159

41 european/ or central european/ or eastern european/ or eu citizen/ or northern european/ or southern european/ or western european/ 146844

42 central european/ or austrian/ or "czech (citizen)"/ or "german (citizen)"/ or "hungarian (citizen)"/ or polish citizen/ or "slovak (citizen)"/ or "slovenian (citizen)"/ or swiss/ 18342

43 eastern european/ or "armenian (citizen)"/ or azerbaijani/ or "belarusian (citizen)"/ or "bulgarian (citizen)"/ or "czech (citizen)"/ or "georgian (citizen)"/ or "hungarian (citizen)"/ or polish citizen/ or "romanian (citizen)"/ or "russian (citizen)"/ or "slovak (citizen)"/ or "ukrainian (citizen)"/ 9113

44 eu citizen/ or austrian/ or belgian/ or "bulgarian (citizen)"/ or "croatian (citizen)"/ or cypriot/ or "czech (citizen)"/ or danish citizen/ or dutchman/ or "estonian (citizen)"/ or "finn (citizen)"/ or frenchman/ or "german (citizen)"/ or "greek (citizen)"/ or "hungarian (citizen)"/ or "irish (citizen)"/ or "italian (citizen)"/ or "latvian (citizen)"/ or "lithuanian (citizen)"/ or "maltese (citizen)"/ or polish citizen/ or "portuguese (citizen)"/ or "romanian (citizen)"/ or "slovak (citizen)"/ or "slovenian (citizen)"/ or spaniard/ or swedish citizen/ 52321

45 northern european/ or british citizen/ or danish citizen/ or "estonian (citizen)"/ or faroese/ or "finn (citizen)"/ or icelander/ or "irish (citizen)"/ or "latvian (citizen)"/ or "lithuanian (citizen)"/ or "norwegian (citizen)"/ or swedish citizen/ 14870

46 british citizen/ or briton/ or northern irish/ 1731

47 briton/ or englishman/ or scotsman/ or welshman/ 1166

48 southern european/ or "albanian (citizen)"/ or "bosnian (citizen)"/ or "croatian (citizen)"/ or cypriot/ or "greek (citizen)"/ or "italian (citizen)"/ or kosovar/ or "macedonian (citizen)"/ or "maltese (citizen)"/ or "portuguese (citizen)"/ or "serbian (citizen)"/ or "slovenian (citizen)"/ or spaniard/ or yugoslav/ 18026

49 western european/ or austrian/ or belgian/ or dutchman/ or frenchman/ or "german (citizen)"/ or swiss/ 22080

For peer review only

50 north american/ or american/ or canadian/ 173924

51 oceanian/ or australian/ or melanesian/ or micronesian/ or new zealander/ or polynesian/ 16781

52 melanesian/ or fijian/ or new caledonian/ or papua new guinean/ 276

53 micronesian/ or marshallese/ or nauruan/ or palauan/ 94

54 polynesian/ or american samoan/ or cook islander/ or french polynesian/ or "hawaiian (citizen)"/ or tongan/ or western samoan/ 406

55 south american/ or argentinian/ or bolivian/ or brazilian/ or chilean/ or colombian/ or ecuadorean/ or guianese/ or guyanese/ or paraguayan/ or peruvian/ or surinamese/ or uruguayan/ or venezuelan/ 17847

56 ethnic group/ or afro-asiatic people/ or altaic people/ or amerind people/ or amish/ or australian aborigine/ or austroasiatic people/ or austronesian people/ or "basque (people)"/ or caucasian speaking people/ or dravidian people/ or eskimo-aleut people/ or "hadza (people)"/ or hmong-mien people/ or indo-european people/ or khoisan/ or na-dene people/ or negrito/ or niger-congo people/ or nilo-saharan people/ or paleosiberian people/ or papuan people/ or pygmy/ or sino-tibetan people/ or tai-kadai people/ or uralic people/ 85845

57 ethnic group/ or exp afro-asiatic people/ or exp altaic people/ or exp amerind people/ or exp amish/ or exp australian aborigine/ or exp austroasiatic people/ or exp austronesian people/ or exp "basque (people)"/ or exp caucasian speaking people/ or exp dravidian people/ or exp eskimo-aleut people/ or exp "hadza (people)"/ or exp hmong-mien people/ or exp indo-european people/ or exp khoisan/ or exp na-dene people/ or exp negrito/ or exp niger-congo people/ or exp nilo-saharan people/ or exp paleosiberian people/ or exp papuan people/ or exp pygmy/ or exp sino-tibetan people/ or exp tai-kadai people/ or exp uralic people/ 190016

58 ethnic minorit*.mp. [mp=title, abstract, heading word, drug trade name, original title, device manufacturer, drug manufacturer, device trade name, keyword heading word, floating subheading word, candidate term word] 20056

59 racial minorit*.mp. [mp=title, abstract, heading word, drug trade name, original title, device manufacturer, drug manufacturer, device trade name, keyword heading word, floating subheading word, candidate term word] 2662

60 multi-ethnic*.mp. [mp=title, abstract, heading word, drug trade name, original title, device manufacturer, drug manufacturer, device trade name, keyword heading word, floating subheading word, candidate term word] 9364

61 BAME.mp. [mp=title, abstract, heading word, drug trade name, original title, device manufacturer, drug manufacturer, device trade name, keyword heading word, floating subheading word, candidate term word] 595

62 BIPOC.mp. [mp=title, abstract, heading word, drug trade name, original title, device manufacturer, drug manufacturer, device trade name, keyword heading word, floating subheading word, candidate term word] 332

63 17 or 18 or 19 or 20 or 21 or 22 or 23 or 24 or 25 or 26 or 27 or 28 or 29 or 30 or 31 or 32 or 33 or 34 or 35 or 36 or 37 or 38 or 39 or 40 or 41 or 42 or 43 or 44 or 45 or 46 or 47 or 48 or 49 or 50 or 51 or 52 or 53 or 54 or 55 or 56 or 57 or 58 or 59 or 60 or 61 or 62 1199105

For peer review only

| | | |
|---|---|---|
| 64 | 16 and 63 | 6820 |

For peer review only

**PsycInfo**

APA PsycInfo <1806 to March Week 3 2023>

1 eating disorders/ or anorexia nervosa/ or "avoidant/restrictive food intake disorder"/ or binge eating disorder/ or bulimia/ or feeding disorders/ or hyperphagia/ or kleine levin syndrome/ or orthorexia/ or pica/ or "purging (eating disorders)"/ or "rumination (eating)"/ or binge eating/ or food addiction/ 36303

2 (feeding and eating disorders).mp. [mp=title, abstract, heading word, table of contents, key concepts, original title, tests & measures, mesh word] 9219

3 diabulimia.mp. 12

4 (feeding and eating disorders of childhood).mp. [mp=title, abstract, heading word, table of contents, key concepts, original title, tests & measures, mesh word] 229

5 night eating syndrome.mp. 267

6 relative energy deficiency in sport.mp. 6

7 eating disorder*.mp. 35849

8 anorexi*.mp. 19945

9 bulimi*.mp. 14423

10 ARFID.mp. 243

11 avoidant restrictive food intake disorder.mp. 334

12 binge eating.mp. 7879

13 pica.mp. 736

14 rumination disorder.mp. 80

15 rumination syndrome.mp. 27

16 1 or 2 or 3 or 4 or 5 or 6 or 7 or 8 or 9 or 10 or 11 or 12 or 13 or 14 or 15 50512

17 "racial and ethnic groups"/ or african cultural groups/ or asians/ or blacks/ or caribbean cultural groups/ or european cultural groups/ or indigenous populations/ or "latinos/latinas"/ or "middle eastern and north african cultural groups"/ or multiracial/ or "people of color"/ or romanies/ or whites/ or cross cultural differences/ or cultural sensitivity/ or ethnic diversity/ or ethnic values/ or multiculturalism/ or "race (anthropological)"/ or "racial and ethnic attitudes"/ or "racial and ethnic differences"/ or racial disparities/ or racial identity/ or systemic racism/ 206179

18 asians/ or chinese cultural groups/ or japanese cultural groups/ or korean cultural groups/ or south asian cultural groups/ or southeast asian cultural groups/ or vietnamese cultural groups/ 28904

19 japanese cultural groups/ or japanese americans/ 1847

20 southeast asian cultural groups/ or vietnamese cultural groups/ 2159

For peer review only

21 indigenous populations/ or alaska natives/ or american indians/ or inuit/ or pacific islanders/ 15920

22 pacific islanders/ or hawaii natives/ 1152

23 "latinos/latinas"/ or mexican americans/ 33166

24 "middle eastern and north african cultural groups"/ or arabs/ 3975

25 whites/ or anglos/ 21972

26 immigration/ or undocumented immigration/ or expatriates/ or human migration/ or migrant workers/ or refugees/ 41412

27 ethnic minorit*.mp. [mp=title, abstract, heading word, table of contents, key concepts, original title, tests & measures, mesh word] 14105

28 racial minorit*.mp. [mp=title, abstract, heading word, table of contents, key concepts, original title, tests & measures, mesh word] 2386

29 multi-ethnic*.mp. [mp=title, abstract, heading word, table of contents, key concepts, original title, tests & measures, mesh word] 1570

30 BAME.mp. [mp=title, abstract, heading word, table of contents, key concepts, original title, tests & measures, mesh word] 121

31 BIPOC.mp. [mp=title, abstract, heading word, table of contents, key concepts, original title, tests & measures, mesh word] 222

32 people of colo*r.mp. [mp=title, abstract, heading word, table of contents, key concepts, original title, tests & measures, mesh word] 2859

33 17 or 18 or 19 or 20 or 21 or 22 or 23 or 24 or 25 or 26 or 27 or 28 or 29 or 30 or 31 or 32 269191

34 16 and 33 1587

For peer review only

**CINAHL**

Accessibility Information and Tips

**Print Search History**

EBSCOhost

| # | Query | Limiters/Expanders | Last Run Via | Results |
|---|---|---|---|---|
| S6 | S1 AND S5 | Expanders - Apply equivalent subjects<br>Search modes - Find all my search terms | Interface - EBSCOhost Research Databases<br>Search Screen - Advanced Search<br>Database - CINAHL | 1,018 |
| S5 | S2 OR S3 OR S4 | Expanders - Apply equivalent subjects<br>Search modes - Find all my search terms | Interface - EBSCOhost Research Databases<br>Search Screen - Advanced Search<br>Database - CINAHL | 186,914 |
| S4 | ethnic minority or racial minority or BAME or BIPOC | Expanders - Apply equivalent subjects<br>Search modes - Boolean/Phrase | Interface - EBSCOhost Research Databases<br>Search Screen - Advanced Search<br>Database - CINAHL | 10,473 |
| S3 | (MH "Immigrants") OR (MH "Undocumented Immigrants") OR (MH "Transients and Migrants") | Expanders - Apply equivalent subjects<br>Search modes - Boolean/Phrase | Interface - EBSCOhost Research Databases<br>Search Screen - Advanced Search<br>Database - CINAHL | 23,520 |
| S2 | (MH "Ethnic Groups+") OR (MH "Amish") OR (MH "Arabs") OR (MH "Asians") OR (MH "Cambodians") OR (MH "Chinese") OR (MH "Filipinos") OR (MH "Hmong") OR (MH "Japanese") OR (MH "Koreans") OR (MH "Laotians") OR (MH "Thais") OR (MH "Vietnamese") OR (MH "Black Persons") OR (MH "African Americans") OR (MH "Roma") OR (MH "Hispanic Americans") OR (MH "Mexican Americans") OR (MH "Indigenous Peoples") OR (MH "Aboriginal Canadians") OR (MH "First Nations of Canada") OR (MH "Arctic Peoples") OR (MH "Inuit") OR (MH "First Nations of Australia") OR (MH "Aboriginal Australians") OR (MH "Torres Strait Islanders") OR (MH "Maori") OR (MH "Native Americans") OR (MH "Alaska Natives") OR (MH "Jews") OR | Expanders - Apply equivalent subjects<br>Search modes - Boolean/Phrase | Interface - EBSCOhost Research Databases<br>Search Screen - Advanced Search<br>Database - CINAHL | 166,196 |

For peer review only

| | | | | |
|---|---|---|---|---|
| | (MH "Kurds") OR (MH "White Persons") | | | |
| S1 | (MH "Eating Disorders+") OR (MH "Eating Disorders Management (Iowa NIC)") OR (MH "Binge Eating Disorder") OR (MH "Feeding and Eating Disorders of Childhood+") OR (MH "Bulimia Nervosa") OR (MH "Avoidant Restrictive Food Intake Disorder") OR (MH "Bulimia") OR "eating disorders or anorexia or bulimia or disordered eating or binge eating disorder | Expanders - Apply equivalent subjects<br>Search modes - Boolean/Phrase | Interface - EBSCOhost Research Databases<br>Search Screen - Advanced Search<br>Database - CINAHL | 29,448 |

For peer review only

**Web of Science**

**(TS=("eating disorder" OR anorexia OR bulimia OR "binge eating" OR pica OR "rumination disorder" OR "disordered eating" OR "eating disorders" OR ARFID OR "avoidant restrictive food intake disorder")) AND TS=(ethnicity OR "ethnic group" OR ethnic OR african OR asian OR black OR caribbean OR "central american" OR european OR  "middle eastern" OR "north american" OR latino OR oceanian OR australian OR white OR caucasian OR immigrant OR migrant OR refugee OR "racial group" OR "racial minority" OR "ethnic minority" OR multi-ethnic OR BAME OR BIPOC )**

**4993 results**

For peer review only

## Preferred Reporting Items for Systematic reviews and Meta-Analyses extension for Scoping Reviews (PRISMA-ScR) Checklist

| SECTION | ITEM | PRISMA-ScR CHECKLIST ITEM | REPORTED ON PAGE # |
|---|---|---|---|
| **TITLE** | | | |
| Title | 1 | Identify the report as a scoping review. | |
| **ABSTRACT** | | | |
| Structured summary | 2 | Provide a structured summary that includes (as applicable): background, objectives, eligibility criteria, sources of evidence, charting methods, results, and conclusions that relate to the review questions and objectives. | |
| **INTRODUCTION** | | | |
| Rationale | 3 | Describe the rationale for the review in the context of what is already known. Explain why the review questions/objectives lend themselves to a scoping review approach. | |
| Objectives | 4 | Provide an explicit statement of the questions and objectives being addressed with reference to their key elements (e.g., population or participants, concepts, and context) or other relevant key elements used to conceptualize the review questions and/or objectives. | |
| **METHODS** | | | |
| Protocol and registration | 5 | Indicate whether a review protocol exists; state if and where it can be accessed (e.g., a Web address); and if available, provide registration information, including the registration number. | |
| Eligibility criteria | 6 | Specify characteristics of the sources of evidence used as eligibility criteria (e.g., years considered, language, and publication status), and provide a rationale. | |
| Information sources* | 7 | Describe all information sources in the search (e.g., databases with dates of coverage and contact with authors to identify additional sources), as well as the date the most recent search was executed. | |
| Search | 8 | Present the full electronic search strategy for at least 1 database, including any limits used, such that it could be repeated. | |
| Selection of sources of evidence† | 9 | State the process for selecting sources of evidence (i.e., screening and eligibility) included in the scoping review. | |
| Data charting process‡ | 10 | Describe the methods of charting data from the included sources of evidence (e.g., calibrated forms or forms that have been tested by the team before their use, and whether data charting was done independently or in duplicate) and any processes for obtaining and confirming data from investigators. | |
| Data items | 11 | List and define all variables for which data were sought and any assumptions and simplifications made. | |
| Critical appraisal of individual sources of evidence§ | 12 | If done, provide a rationale for conducting a critical appraisal of included sources of evidence; describe the methods used and how this information was used in any data synthesis (if appropriate). | |
| Synthesis of results | 13 | Describe the methods of handling and summarizing the data that were charted. | |

For peer review only

| SECTION | ITEM | PRISMA-ScR CHECKLIST ITEM | REPORTED ON PAGE # |
|---|---|---|---|
| **RESULTS** | | | |
| Selection of sources of evidence | 14 | Give numbers of sources of evidence screened, assessed for eligibility, and included in the review, with reasons for exclusions at each stage, ideally using a flow diagram. | |
| Characteristics of sources of evidence | 15 | For each source of evidence, present characteristics for which data were charted and provide the citations. | |
| Critical appraisal within sources of evidence | 16 | If done, present data on critical appraisal of included sources of evidence (see item 12). | |
| Results of individual sources of evidence | 17 | For each included source of evidence, present the relevant data that were charted that relate to the review questions and objectives. | |
| Synthesis of results | 18 | Summarize and/or present the charting results as they relate to the review questions and objectives. | |
| **DISCUSSION** | | | |
| Summary of evidence | 19 | Summarize the main results (including an overview of concepts, themes, and types of evidence available), link to the review questions and objectives, and consider the relevance to key groups. | |
| Limitations | 20 | Discuss the limitations of the scoping review process. | |
| Conclusions | 21 | Provide a general interpretation of the results with respect to the review questions and objectives, as well as potential implications and/or next steps. | |
| **FUNDING** | | | |
| Funding | 22 | Describe sources of funding for the included sources of evidence, as well as sources of funding for the scoping review. Describe the role of the funders of the scoping review. | |

JBI = Joanna Briggs Institute; PRISMA-ScR = Preferred Reporting Items for Systematic reviews and Meta-Analyses extension for Scoping Reviews.

* Where *sources of evidence* (see second footnote) are compiled from, such as bibliographic databases, social media platforms, and Web sites.

† A more inclusive/heterogeneous term used to account for the different types of evidence or data sources (e.g., quantitative and/or qualitative research, expert opinion, and policy documents) that may be eligible in a scoping review as opposed to only studies. This is not to be confused with *information sources* (see first footnote).

‡ The frameworks by Arksey and O'Malley (6) and Levac and colleagues (7) and the JBI guidance (4, 5) refer to the process of data extraction in a scoping review as data charting.

§ The process of systematically examining research evidence to assess its validity, results, and relevance before using it to inform a decision. This term is used for items 12 and 19 instead of "risk of bias" (which is more applicable to systematic reviews of interventions) to include and acknowledge the various sources of evidence that may be used in a scoping review (e.g., quantitative and/or qualitative research, expert opinion, and policy document).

*From:* Tricco AC, Lillie E, Zarin W, O'Brien KK, Colquhoun H, Levac D, et al. PRISMA Extension for Scoping Reviews (PRISMAScR): Checklist and Explanation. Ann Intern Med. 2018;169:467–473. doi: 10.7326/M18-0850.

For peer review only
